# METTL3 promotes chemoresistance in small cell lung cancer by inducing mitophagy

**DOI:** 10.1186/s13046-023-02638-9

**Published:** 2023-03-17

**Authors:** Yueqin Sun, Weitao Shen, Shulu Hu, Qiong Lyu, Qiongyao Wang, Ting Wei, Weiliang Zhu, Jian Zhang

**Affiliations:** Department of Oncology, Zhujiang Hospital, Southern Medical University, Guangzhou, China

**Keywords:** Small cell lung cancer, Chemoresistance, N6-methyladenosine, Mitophagy

## Abstract

**Background:**

Small cell lung cancer (SCLC) is the most aggressive subtype of lung cancer. Although most patients are initially sensitive to first-line combination chemotherapy with cisplatin and etoposide, chemotherapy drug resistance easily develops and quickly leads to tumour progression. Therefore, understanding the mechanisms of chemotherapy drug resistance and how to reverse it is key to improving the prognosis of patients with SCLC. Moreover, N6-methyladenosine (m6A) is the most abundant mRNA modification and is catalysed by the methyltransferase complex, in which methyltransferase-like 3 (METTL3) is the sole catalytic subunit.

**Methods:**

The effects of METTL3 on chemoresistance in SCLC cells were determined using qRT–PCR, Western blotting, immunohistochemistry, cell counting kit (CCK-8) assays, flow cytometry, and tumorigenicity experiments. Methylated RNA immunoprecipitation sequencing (MeRIP-seq), MeRIP qPCR, immunofluorescence, and drug inhibitor experiments were performed to confirm the molecular mechanism of Decapping Protein 2 (DCP2), which is involved in the chemoresistance of SCLC.

**Results:**

In the present study, we found that METTL3 is a marker for poor SCLC prognosis, and it is highly expressed in chemoresistant SCLC cells. METTL3 promotes SCLC chemoresistance by positively regulating mitophagy. METTL3 induces m6A methylation of DCP2 and causes the degradation of DCP2, which promotes mitochondrial autophagy through the Pink1-Parkin pathway, leading to chemotherapy resistance. We also found that STM2457, a novel METTL3 inhibitor, can reverse SCLC chemoresistance.

**Conclusions:**

The m6A methyltransferase METTL3 regulates Pink1-Parkin pathway-mediated mitophagy and mitochondrial damage in SCLC cells by targeting DCP2, thereby promoting chemotherapy resistance in patients with SCLC.

**Supplementary Information:**

The online version contains supplementary material available at 10.1186/s13046-023-02638-9.

## Background

Lung cancer has the highest worldwide incidence and mortality rates of all cancer types, and its 5-year overall survival rate is poor (less than 20%) [1, 2]. Lung cancer can be divided into two subtypes according to its pathological characteristics: small cell lung cancer (SCLC) and non-small cell lung cancer [3, 4]. SCLC has caused many deaths due to its strong invasiveness, high degree of malignancy, and potential to develop drug resistance. Initially, most SCLC patients respond to first-line platinum-containing chemotherapy. However, patient responses are often not long-lasting, and it is easy to develop chemotherapy resistance, resulting in disease recurrence [5, 6]. Therefore, exploring the chemoresistance mechanisms of SCLC is crucial to identify therapeutic targets that can reverse it.

N6 adenylate methylation (m6A) is the most abundant eukaryotic mRNA modification [7]. The m6A modification regulates mRNA processing, and it is dynamically and reversibly mediated by methyltransferases (writers), demethylases (erasers) and m6A binding proteins (readers). METTL3, a “writer” for m6A, is one of the most important m6A methyltransferases and is involved in proliferation, migration, and other biological processes [8, 9]. Over the past few years, as the understanding of m6A modification has advanced, it has been reported that m6A is involved in drug resistance in various solid tumours, including liver cancer, colon cancer, and gastric cancer [10–12].However, the relationship between METTL3-mediated m6A methylation and chemoresistance in SCLC has not been explored.

All eukaryotic mRNA transcripts have a 7-methylguanosine cap at the 5′ end, which is essential for precursor mRNA processing, translation initiation, and mass mRNA decay. Therefore, decapping is a key regulatory process in the life cycle of eukaryotic mRNAs because it allows for the degradation of transcripts and controls a number of inputs [13–15]. DCP2 is the major decapping enzyme during 5′ to 3′ mRNA decay. DCP2 cleaves the 5′-capped mRNA between the α- and β-phosphate moieties, releasing 7-methylguanosine diphosphate molecules and a 5′-monophosphorylated mRNA, which in turn regulate mRNA stability [16–18]. Although DCP2 is one of the most important decapping enzymes, it is not known whether DCP2 is involved in chemotherapy resistance. In the present study, we found that DCP2 is a downstream target of METTL3 and that DCP2 m6A methylation by METTL3 promotes SCLC chemotherapy resistance.

Mitophagy is a type of selective autophagy that is very important for mitochondrial quality control [19]. Under conditions such as reactive oxygen species stimulation, nutrient deprivation, and cellular aging, mitochondria undergo membrane potential depolarization. Cellular proteins and depolarized mitochondria are sequestered in autophagosomes, which subsequently fuse with lysosomes and are degraded, maintaining the stability of the intracellular environment and mitochondrial fitness [20–22]. By sensing different extracellular signals, mitochondrial autophagy is involved in a variety of biological processes, such as mitochondrial depolarization, hypoxia, development [23–25]. Previous studies have shown that autophagy is involved in SCLC resistance to chemotherapy, and autophagy is significantly enhanced in chemoresistant SCLC cell lines [26, 27]. However, there are few reports on whether mitochondrial autophagy specifically is associated with SCLC resistance.

In the present study, we found that METTL3 was highly expressed in chemoresistant SCLC cell lines and promoted SCLC chemoresistance. In addition, high METTL3 expression was associated with poor SCLC patient prognosis. The results of MeRIP-seq and RNA-seq showed that DCP2 was the downstream target of METTL3, and METTL3 induced m6A methylation of DCP2, which further promoted mitochondrial autophagy and affected the chemosensitivity of SCLC cells. To summarize, our study reveals the relationships between m6A modification, chemoresistance, and mitochondrial autophagy, which furthers our understanding of SCLC chemoresistance mechanisms. Our findings also suggest that METTL3 may be a therapeutic target for reversing chemotherapy resistance in SCLC.

## Materials and methods

### Tissue sample collection

From September 2009 to September 2018, 58 fresh-frozen tissue samples were collected from SCLC patients. The patients received care and follow-up at Zhujiang Hospital (Southern Medical University, Guangzhou, China), the First Affiliated Hospital of Guangzhou Medical University (Guangzhou, China), the Collaborative Innovation Center for Cancer Medicine (Guangzhou, China), or the Nangfang Hospital (Southern Medical University, Guangzhou, China). Informed consent was obtained from all patients before specimen collection. The experiments were approved by the Ethics Committee of Southern Medical University (Guangzhou, China). The patient clinical information is detailed in Table S1.

### Data set

To evaluate the effect of METTL3 expression on the survival prognosis of SCLC, we performed univariate Cox regression analysis and survival analysis on GSE60052, a dataset from the Gene Expression Omnibus (GEO) database (https://www.ncbi.nlm.nih.gov/geo/query/acc.cgi?acc=GSE60052), which contains whole genome sequencing data and clinical information from 79 patients with SCLC [28].

### Cell lines

The SCLC cell lines H69, H69AR (a cell line induced by H69 with doxorubicin), H446, and HBE were purchased from ATCC (American Type Culture Collection, USA). Multidrug-resistant cells, H446DDP, were developed by incubating H446 cells with increasing doses of cisplatin (up to 5 μg/mL) for 6 months, as described in our previous study [29]. The chemoresistant cell lines H69AR and H446DDP were cultured with the concentrations of cisplatin and adriamycin necessary to maintain their chemoresistance.

### RNA isolation and real-time qRT-PCR

Total RNA was extracted from SCLC cells using TRIzol reagent (Invitrogen) according to the manufacturer’s instructions. A Nanodrop 2000 instrument (Thermo Scientific) was used to measure RNA concentrations. qRT–PCR was performed with an ABI Illumina instrument (Foster, USA) using SYBR Green (Tiangen). The relative mRNA expression levels were obtained using the 2^−ΔΔCT^ method. All primer sequences are shown in Table S2.

### RNA sequencing, data processing and gene difference analysis of SCLC cells

RNA was extracted from SCLC cells (H69 and H69AR). The RNA quality was assessed with a Bioanalyzer 2100 DNA Chip 7500 instrument (Agilent Technologies), and samples with an RNA integrity number (RIN) of over 7 were further analysed by RNA-seq. All sequencing reactions were performed on an Illumina HiSeq 2000 instrument (Illumina, San Diego, CA, USA). We used HISAT2 (version 2.1.0) with the default setting to map the RNA-seq data to the human reference genome (NCBI38/hg38). We aggregated the read counts at the gene level using HTSeq. The R package ‘edgeR’ was used to analyse the difference in gene expression data (raw count), and the R package ‘complexHeatmap’ was used to visualize the results [30, 31].

### Western blot analysis

SCLC cells were collected, washed with cold PBS, and incubated with RIPA lysis buffer (Biyuntian) at 4 °C for 30 minutes to extract the total protein. Protein concentrations were determined by a BCA protein assay, equal protein was loaded and run on an SDS–PAGE gel, and gel contents were transferred to a PVDF membrane. The membrane was sealed with 5% bovine serum albumin (BSA) powder, incubated in primary antibody solution at 4 °C overnight, and then incubated with horseradish peroxidase-coupled secondary antibody for 1-2 hours. Finally, proteins were detected by chemiluminescence (ECL). Information on the antibodies used in this study is shown in Table S3.

### Transfection

Cells were transiently transfected with DCP2 siRNA (Gene Pharma, Shanghai, China) using Lipofectamine 3000 and OPTI-MEM (Invitrogen, USA) according to the manufacturer’s instructions. For stable expression, lentivirus encoding shMETTL3, noncoding shRNA (shNC), overexpression METTL3 (OE-METTL3), or OE-NC (GeneChem) was transduced into SCLC cells. Forty-eight hours after infection, 2.0 μg/mL puromycin (Solarbio) was used to select for successfully infected cells. The infection efficiency was verified by qRT–PCR and Western blotting. The sequences of the shRNAs and siRNAs are shown in Tables S4 and S5.

### Cell counting kit-8 (CCK-8) assay

A cell counting kit-8 (CCK8) assay was used to analyse cell proliferation and drug resistance. After transient transfection or stable transduction was confirmed, the cells were seeded into 96-well plates at approximately 5 × 10^3^ cells per well and then incubated with different concentrations of cisplatin (CDDP, Shandong, China), etoposide (VP16, Jiangsu, China), and STM2457 (MedChemExpress, China) for 24 hours. Cells were treated for 3 h with 10 μL CCK-8 reagent (Dojindo), and the absorbance values at 450 nm were recorded. The IC50 of each chemotherapy drug was calculated according to the OD value. For each condition, five replicate wells were plated, and three parallel experiments were conducted.

### Flow cytometry

Transfected cells were treated with chemotherapeutic drugs (CDDP and VP16) and a METTL3 inhibitor (STM2457) for 24 hours to induce apoptosis, and cells were collected for experiments. The dosage of chemotherapeutic drugs was equivalent to 1/2-1/3 of the IC50 of SCLC cells. Apoptosis was assessed using the Annexin V-FITC Apoptosis Detection Kit (BestBio, Shanghai, China).

### In vivo study

The BALB/C nude mice used in this study were purchased from the Experimental Animal Center of Southern Medical University (Guangzhou, China) and raised in a specific pathogen-free (SPF) environment. SCLC cells were collected, and cell suspensions were prepared at a concentration of 1 × 10^6^ cells per 100 μL PBS and injected subcutaneously into nude mice. When the tumour volume reached 60 mm^3^, mice were randomly divided into control and treatment groups with five mice in each group. The mice in the treatment group were treated regularly, and chemotherapy drugs (CDDP 3 mg/kg and VP16 2 mg/kg) were administered via intraperitoneal injection. The health status of the mice and growth of the subcutaneous tumour were observed every 2-3 days. The tumour size was measured with callipers, and the tumour volume was calculated according to the following standard formula: V = (Length×Width^2^/ 2).

### Immunohistochemistry

Tissue samples from nude mice with subcutaneous SCLC tumours were fixed in 4% paraformaldehyde and embedded in paraffin blocks. The samples were treated with specific antibodies against METTL3 (Abcam) and DCP2 (Abcam) overnight at 4 °C. Then, the samples were incubated with a secondary antibody for 2 hours. The results were visualized using the EnVision Peroxidase System (Dako).

### Detection of mitochondrial autophagy and mitochondrial membrane potential

Mitophagy in live cells was monitored by Dojindo Molecular Technologies. Mitochondrial autophagy was induced by chemotherapeutic drug treatments. The level of mitochondrial autophagy was defined by the Mtphagy dye area of each cell. The colocalization of Mtphagy and lysosomal dyes was also analysed. JC-1 staining (Beyotime Biotechnology) was used to detect mitochondrial membrane potential, and all samples were observed by laser confocal microscopy (Carl Zeiss, lsm710, Carl Zeiss, Oberkochen, Germany).

### Immunofluorescence

Cells were seeded on a confocal dish, fixed with 4% paraformaldehyde, permeabilized with 0.3% Triton X-100, sealed with 5% BSA for 2 hours, and incubated overnight with the appropriate primary antibody. Then, the cells were incubated with allogeneic secondary antibody in the dark for 1 hour. Finally, nuclei were stained with DAPI, and images were collected by confocal microscopy.

### Methylated RNA immunoprecision sequencing (MeRIP-seq) and MeRIP qPCR

These methods were adapted from previous literature [32]. Using the riboMeRip m6A Transcript Profiling Kit (RiboBio, China), 100 μl of poly(A) RNA was purified from total RNA, and one tenth of the total RNA was used as the input control. Magnetic beads A/G were prewashed and incubated with an anti-m6A antibody for 2 hours at 4 °C. After three washes, the RNA was collected. The eluted RNA was then purified and recovered using the Magen Hipure Serum/Plasma miRNA Kit (Magen, China) for further MeRIP sequencing. The m6A modifications of individual genes were determined using the MeRIP-qPCR assay. In brief, primers were designed against the methylation sites of individual genes based on MeRIP-seq results (Table S2). The RNA recovered after IP purification was reverse transcribed and subjected to qPCR enrichment analysis, and the corresponding m6A enrichment in each sample was calculated by normalization to the input data.

### Mitochondrial protein extraction and detection

Mitochondrial protein extraction from SCLC cells was performed with a Mitochondria Isolation Kit for Cultured Cells (Thermo Fisher Scientific) according to the manufacturer’s instructions. Then, the mitochondrial proteins were analysed by Western blotting.

### Mitochondrial ROS (mROS) measurement

mROS were detected using a MitoSOX Red mitochondrial superoxide indicator (YEASEN, Shanghai, China) according to the manufacturer’s instructions. SCLC cells were added to confocal dishes. SCLC cells were incubated with 5 μM of MitoSOX reagent working solution for 10 min at 37 °C in the dark. After washing three times with warm PBS, mROS were observed by laser confocal microscopy (Carl Zeiss, Oberkochen, Germany).

### Statistical analysis

All data in this study were statistically analysed using GraphPad Prism 7 and are expressed as the mean ± standard deviation (SD). All experiments were independently conducted three times. *P* < 0.05, *P* < 0.01, and *P* < 0.001 were considered statistically significant and are denoted by “*”, “**”, and “***”, respectively.

## Results

### METTL3 is highly expressed in chemoresistant SCLC cell lines and is associated with poor patient prognosis

**Fig. 1:**
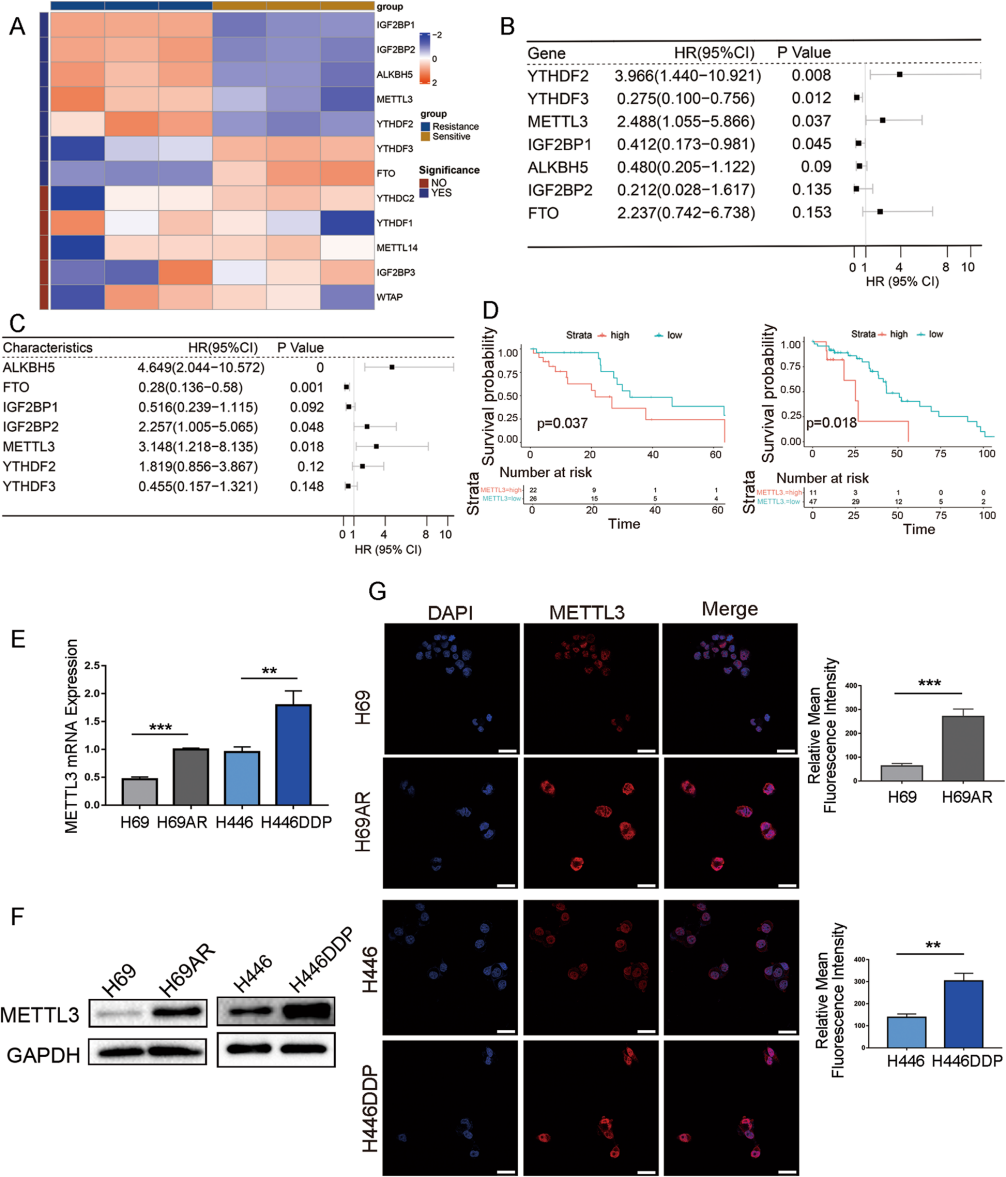
Expression of METTL3 and its clinical significance in SCLC. **A** Heatmap of the mRNA expression of 12 common m6A regulators in the SCLC chemotherapy-sensitive cell line H69 and the drug-resistant cell line H69AR based on whole-transcriptome sequencing. Cell types are indicated on the top of the heatmap, and m6A regulator-specific gene names are indicated on the right; the left side shows significance. If *P* < 0.05, the significance is marked as YES, and if *P* ≥ 0.05, the significance is marked as NO. **B-C** Forest plot showing the univariate Cox regression analysis of significant differences between m6A regulators and overall survival (OS) in the SCLC clinical cohort GSE60052 from the GEO public database (**B**) and the SCLC clinical cohort from local hospitals (**C**). The main part of the forest plot is composed of hazard ratios (HRs) and 95% confidence intervals (CIs), and the *P* value is also marked in the fig. HR < 1 is a predictor for favourable OS, and HR > 1 is a predictor for poor OS. **D** Kaplan–Meier analysis was used to compare the effect of high and low METTL3 expression on OS in the SCLC cohort from GSE60052 (left) and from local hospitals (right); *P* values are marked in the fig. **E** qRT-PCR analysis of METTL3 mRNA expression in two pairs of chemosensitive and chemoresistant SCLC cells, H69/H69AR and H446/H446DDP. **P < 0.01; ***P < 0.001. **F** Western blot analysis of METTL3 expression in two pairs of chemosensitive and chemoresistant SCLC cells, H69/H69AR and H446/H446DDP. **G** Immunofluorescence analysis of METTL3 expression in H69/H69AR and H446/H446DDP cells. Scale bars, 25 μm. Quantification of METTL3 expression detected by immunofluorescence is shown. The data originated from 3 independent experiments. **P < 0.01; ***P < 0.001

Many studies have shown that m6A modification plays a role in the chemotherapy or targeted drug resistance of various solid tumours, such as gastric cancer and liver cancer [10–12]. To determine whether m6A is involved in SCLC chemoresistance, high-throughput whole-transcriptome sequencing of sensitive and resistant SCLC cell lines (H69 and H69AR) was performed, and differences in the expression of genes associated with m6A were analysed. We observed significant differences in the expression of seven genes associated with m6A modification between chemotherapy-sensitive and chemotherapy-resistant cell lines (Fig. 1A). The expression of these seven genes in two SCLC patient cohorts was analysed by univariate Cox regression analysis, and the relationship between the expression of m6A-associated genes and patient prognosis was further analysed. Cohort 1, from the public database GEO (GSE60052), included transcriptomic and clinical data of 79 SCLC patients, and cohort 2 included 58 SCLC patients treated in local hospitals. For the univariate Cox regression analysis of overall survival (OS), only METTL3 was correlated with SCLC survival and prognosis in both cohorts (Fig. 1B-C). Survival curves in both cohort 1 and cohort 2 showed that high METTL3 expression was associated with worse OS and that METTL3 was a marker of poor prognosis (Fig. 1D).

To further clarify whether METTL3 plays a key role in SCLC chemoresistance, qRT–PCR and Western blotting were used to analyse METTL3 expression in two pairs of chemotherapy drug-sensitive and -resistant cell lines (H69 and H69AR; H446 and H446DDP). METTL3 mRNA and protein expression levels were significantly higher in the chemoresistant H69AR and H446DDP cell lines than in their chemosensitive counterparts (Fig. 1E-F). Immunofluorescence also showed that METTL3 was highly expressed in drug-resistant SCLC cell lines and that it mainly localized in the nucleus as well as a small part in the cytoplasm (Fig. 1G).

### Overexpression of METTL3 promotes SCLC chemoresistance in vitro

**Fig. 2:**
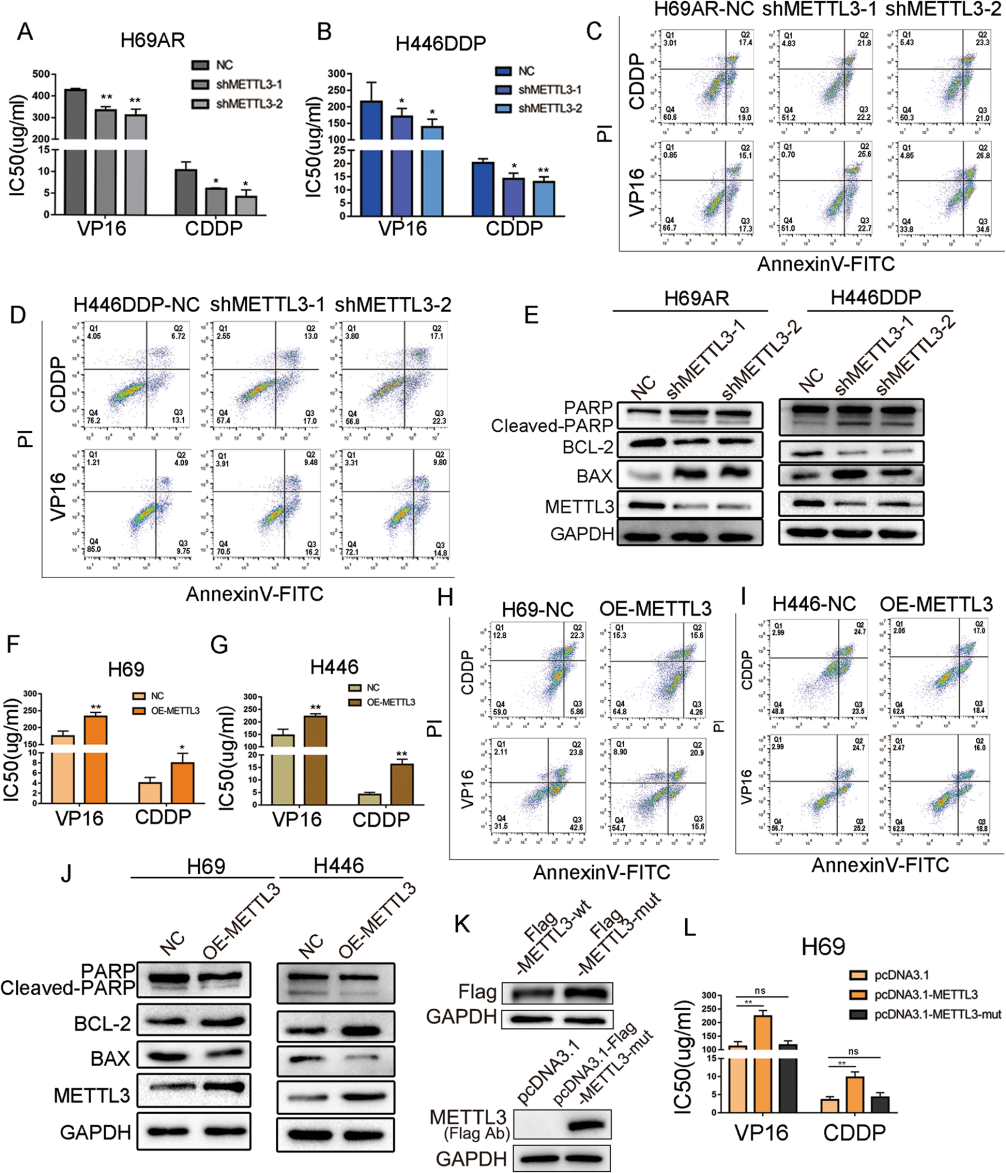
METTL3 induces SCLC chemoresistance in vitro. **A-B** CCK-8 assays showed that knockdown of METTL3 decreased the chemotherapy IC50 values in chemoresistant H69AR and H446DDP cells. *P < 0.05, **P < 0.01. **C-D** Cell apoptosis and arrest were evaluated by flow cytometric analysis in METTL3-knockdown SCLC cells after treatment with chemotherapeutic drugs. **E** Apoptosis-related protein levels were measured by Western blotting following treatment with chemotherapeutic drugs in METTL3 knockdown cells. **F-G** CCK-8 assays showed that METTL3 overexpression increased the IC50 values of chemotherapeutic agents in chemosensitive H69 and H446 cells. *P < 0.05, **P < 0.01. **H-I** Cell apoptosis and arrest were evaluated by flow cytometry in METTL3-overexpressing SCLC cells following treatment with chemotherapeutic drugs. **J** Apoptosis-related proteins were measured by Western blotting following treatment with chemotherapeutic drugs in METTL3-overexpressing cells. **K** Western blot assays were performed to evaluate METTL3 expression after transfection with pcDNA3.1-METTL3 and pcDNA3.1-METTL3-mut in H69 cells. **L** CCK-8 assay showed that overexpression of METTL3 changed the IC50 value of chemotherapy drugs in SCLC cells. **P < 0.01; ns, not significant

To further evaluate whether METTL3 is involved in SCLC chemotherapy resistance, we constructed stable METTL3-knockdown H69AR and H446DDP cell lines using lentivirus transduction. qRT–PCR and Western blotting were performed 48 hours after transduction. The two METTL3 shRNAs substantially decreased METTL3 expression levels in chemoresistant cells (Fig. S1A-B). The IC50 (half maximal inhibitory concentration) and cell apoptosis were examined 24 hours after exposure to chemotherapy, and knockdown of METTL3 in drug-resistant cell lines significantly reduced the IC50 of the chemotherapeutic drugs CDDP and VP16 (Fig. 2A-B). The flow cytometry results also showed a higher proportion of apoptotic cells in the METTL3 knockdown group than in the control group, and this difference was statistically significant (Figs. 2C-D and S1C-D). Western blotting showed that the levels of the apoptotic proteins cleaved PARP and BAX, which indicate the intensity of apoptosis, were significantly increased in the METTL3-knockdown group, while the expression levels of the antiapoptotic protein BCL-2 was significantly decreased (Fig. 2E).

To complement our findings from chemoresistant cell lines, we overexpressed METTL3 in the chemosensitive cell lines H69 and H446. qRT–PCR and Western blot analysis showed that METTL3 was successfully overexpressed in these cells (Fig. S1E-F). As expected, the chemotherapy IC50 values of cells overexpressing METTL3 were significantly increased compared to those of empty vector controls (Fig. 2F-G). Western blot and flow cytometry analysis of apoptotic proteins also showed that METTL3 overexpression significantly reduced the proportion of apoptotic cells (Figs. 2H-J and S1G-H).

To elucidate whether METTL3-induced SCLC chemotherapy resistance depends on its methyltransferase function, we constructed METTL3 wild-type (pcDNA3.1-METTL3) and mutant (pcDNA3.1-METTL3-mut, with methyltransferase domain deletion) recombination plasmids [33, 34] (Fig. 2K). Overexpressing METTL3 in SCLC cells significantly increased the IC50 of chemotherapy drugs, and deletion of the METTL3 methyltransferase domain blocked the chemotherapy resistance induced by METTL3 (Fig. 2L). These data indicate that METTL3 regulates the chemotherapy resistance process in SCLC through its methyltransferase domain.

### METTL3 promotes SCLC chemoresistance in vivo

**Fig. 3:**
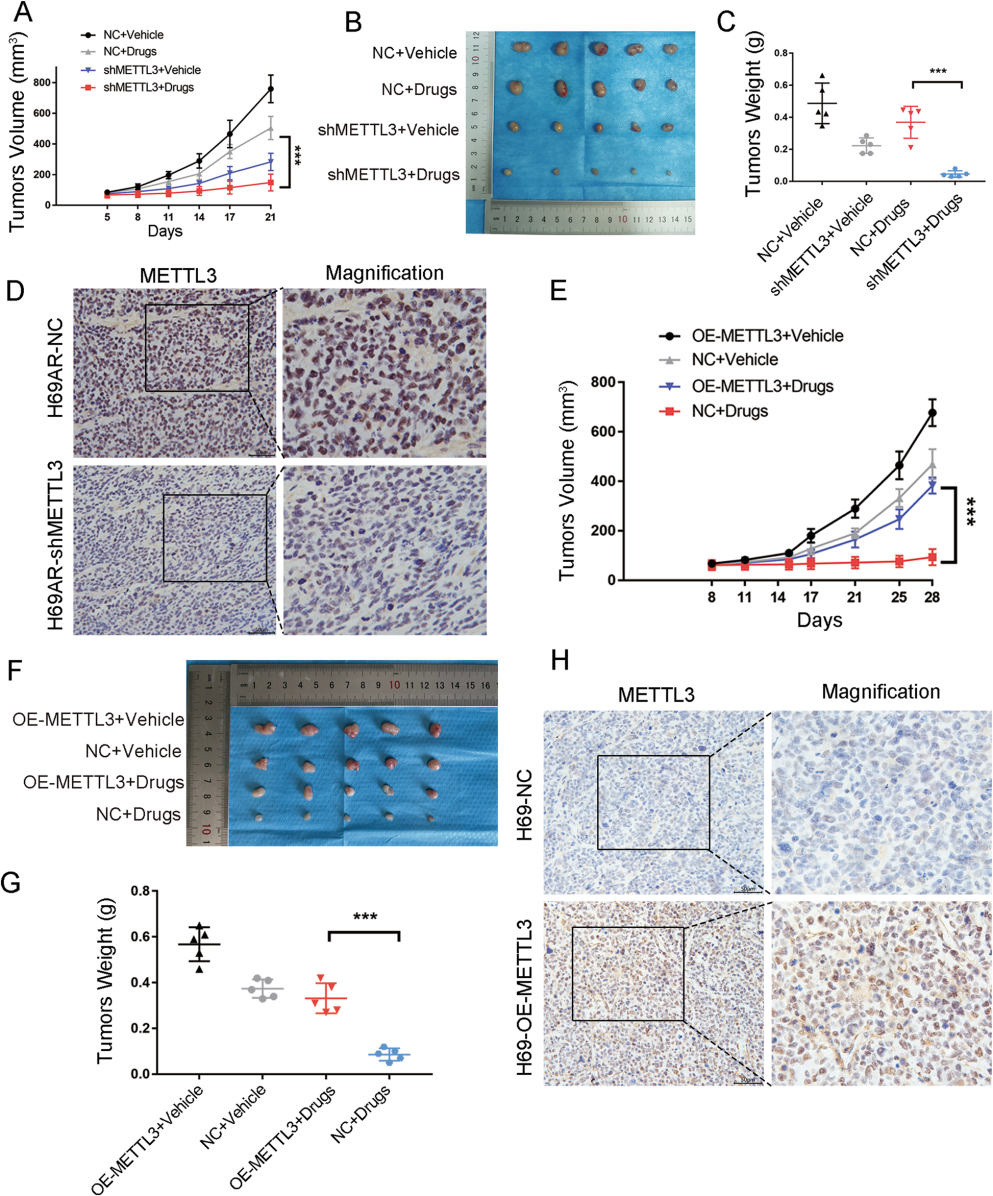
Effect of METTL3 on SCLC chemoresistance in vivo. **A** Tumour growth in the METTL3 knockdown groups was measured. H69AR cells were stably transduced with shNC or shMETTL3. Cells were subcutaneously injected into nude mice, and chemotherapeutic drugs or vehicles were administered intraperitoneally. ***P < 0.001. **B-C** Effect of METTL3 on resistance to chemotherapeutic drugs in nude mice and measurement of the tumour weights ***P < 0.001. **D** Representative IHC staining of METTL3 in subcutaneous xenografts with or without METTL3 knockdown. Scale bars, 50 μm. **E** Tumour growth in the METTL3 overexpression groups was measured. H69 cells stably overexpressing METTL3 or control cells were subcutaneously injected into nude mice, and chemotherapeutic drugs or vehicles were administered intraperitoneally. ***P < 0.001. **F-G** Effect of METTL3 on sensitivity to chemotherapeutic drugs in nude mice and measurement of the tumour weights of different groups. ***P < 0.001. **H** Representative IHC staining of METTL3 in subcutaneous xenografts with or without METTL3 overexpression. Scale bars, 50 μm

Since the platinum–etoposide combination regimen remains the first-line treatment option for SCLC, CDDP and VP16 were simultaneously used to study whether METTL3 is involved in SCLC chemoresistance in vivo. Nude mice were subcutaneously injected with METTL3-knockdown H69AR cells or METTL3-overexpressing H69 cells, and mice were treated with PBS or chemotherapeutic drugs (CDDP 3 mg/kg and VP-16 2 mg/kg). METTL3 knockdown significantly inhibited the growth of the transplanted tumour (Fig. 3A) and significantly reduced the volume of the transplanted tumour following chemotherapy (Fig. 3B-C). METTL3 expression in tumour xenografts was evaluated by IHC (Fig. 3D). In contrast, the growth rate and volume of the METTL3-overexpressing H69 tumours were significantly higher than those of the control H69 tumours (Fig. 3E-G). The expression of METTL3 in the tumour xenografts was also evaluated by IHC (Fig. 3H). These findings suggest that METTL3 can promote SCLC chemotherapy resistance in vivo and that METTL3 could be an effective therapeutic target for reversing SCLC chemoresistance.

### METTL3 promotes the m6A methylation of DCP2 and regulates the expression of DCP2

**Fig. 4:**
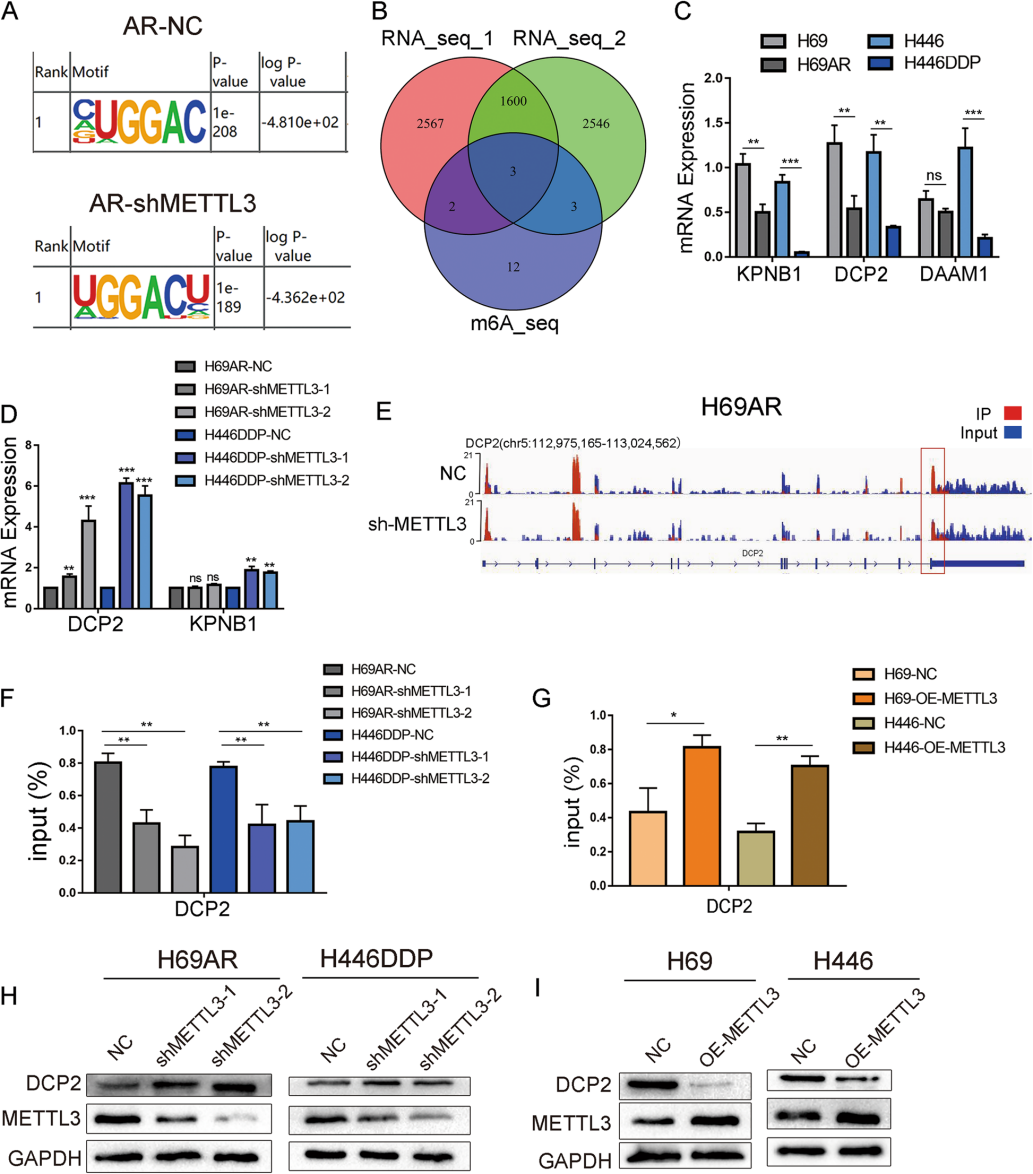
DCP2 is a downstream target of METTL3. **A** Consensus motif map with MeRIP-seq peaks identified by HOMER in the SCLC drug-resistant cell line H69AR after METTL3 knockdown. **B** Venn diagram showing the gene set with significantly decreased m6A levels following METTL3 knockdown as measured by MeRIP-seq and the two gene sets with significant differences in RNA-seq differential expression analysis. The intersection of these gene sets yielded three significantly different genes (DCP2, DAAM1, and KPNB1). **C** qRT-PCR analysis: mRNA expression of DCP2, DAAM1 and KPNB1 in sensitive and resistant cell lines was detected by qRT–PCR. **P < 0.01; ***P < 0.001; ns, not significant. **D** qRT-PCR analysis: mRNA expression of DCP2 and KPNB1 after METTL3 knockdown in the drug-resistant cell lines H69AR and H446DDP. **P < 0.01; ***P < 0.001; ns, not significant. **E** IGV map from MeRIP-seq shows that DCP2 m6A methylation levels were significantly decreased in METTL3-knockdown H69AR cells. **F** MeRIP-qPCR analysis of DCP2 m6A methylation levels in drug-resistant cell lines and their METTL3-knockdown stably transduced counterparts. **P < 0.01. **G** MeRIP-qPCR analysis of DCP2 m6A methylation levels in chemosensitive cell lines and their counterparts that stably overexpress METTL3. *P < 0.05, **P < 0.01. **H** Western blot analysis of DCP2 expression in H69AR and H446DDP cells transfected with shRNA-METTL3. **I** Western blot analysis of DCP2 expression in H69 and H446 cells transfected with METTL3

To further clarify METTL3’s target gene and the mechanisms by which METTL3 contributes to SCLC chemoresistance, we performed MeRIP-seq on METTL3 knockdown and control H69AR cells, and the GGAC motif was highly enriched in the m6A site of both knockdown and control cells (Fig. 4A). Since METTL3 is a m6A methyltransferase, we focused on genes with significantly decreased m6A methylation levels following METTL3 knockdown and selected the top 20 genes by fold difference. Simultaneously, RNA-seq was performed on H446DDP and parental H446 cells in which METTL3 was successfully knocked down or overexpressed. Then, the MeRIP-seq gene set was intersected with the RNA-seq genes with *P* < 0.01 after differential analysis, and three genes were identified as significantly different between the three cohorts (Fig. 4B). To identify downstream target genes of METTL3, we performed fluorescence qPCR on the three distinct intersecting genes and found that DCP2 is a METTL3 target gene in SCLC (Fig. 4C-D). The IGV map from MeRIP-seq also showed that after METTL3 knockdown, m6A methylation levels in the coding region (CDS) of DCP2 was significantly decreased (Fig. 4E). These findings were confirmed by MeRIP-qPCR. In H69AR and H446DDP cells, METTL3 knockdown resulted in significantly decreased DCP2 m6A methylation levels, and in H69 and H446 cells, METTL3 overexpression significantly increased DCP2 m6A methylation levels (Fig. 4F-G).

Western blotting and immunofluorescence showed that DCP2 was highly expressed in H69 and H446 cells (Fig. S2A), mainly in the cytoplasm and in a small area of the nucleus (Fig. S2B-C). METTL3 knockdown resulted in significantly increased DCP2 protein levels, whereas METTL3 overexpression significantly decreased DCP2 protein levels (Fig. 4H-I). Overall, these findings indicate that DCP2 is the target gene of METTL3 and that METTL3 downregulates DCP2 expression by inducing m6A methylation of DCP2.

### DCP2 affects SCLC chemoresistance by regulating mitophagy

**Fig. 5:**
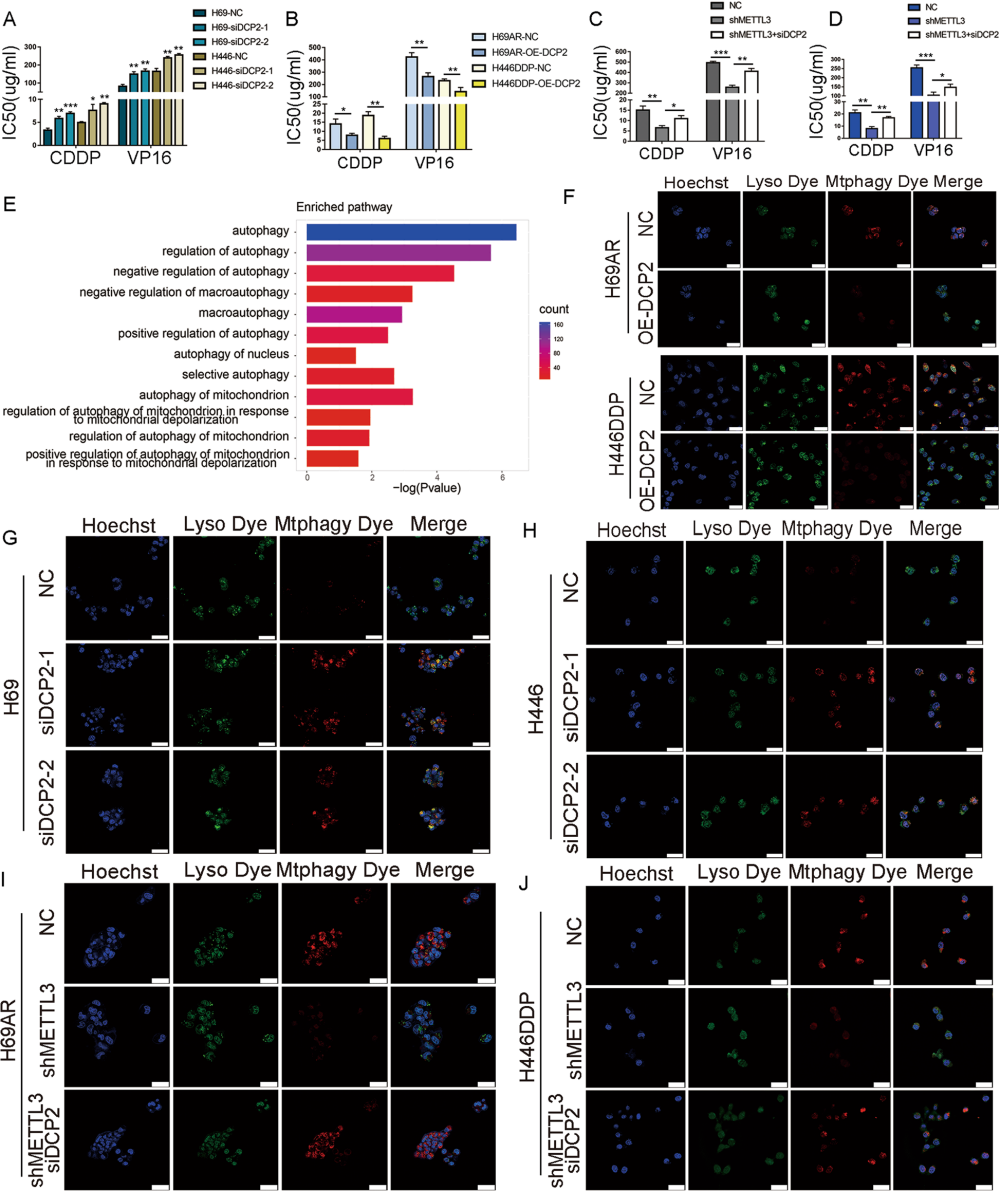
DCP2 prevents SCLC chemoresistance by regulating mitophagy. **A** CCK-8 assays show that knockdown of DCP2 increased the IC50 values of chemotherapeutic drugs for the chemosensitive H69 and H446 cell lines. *P < 0.05; **P < 0.01; ***P < 0.001. **B** CCK-8 assays show that DCP2 overexpression decreased the IC50 values of chemotherapeutic agents for the chemoresistant H69AR and H446DDP cell lines. *P < 0.05; **P < 0.01. **C-D** CCK-8 assays showing the effect of simultaneous METTL3 and DCP2 knockdown on the IC50 of chemotherapeutic drugs for H69AR and H446DDP cells. *P < 0.05; **P < 0.01; ***P < 0.001. **E** Histogram showing the significantly enriched autophagy-related pathways after GO pathway enrichment analysis of MeRIP-seq data. **F** Mtphagy images show mitophagy fluorescence, and merged images show colocalization between mitochondrial and lysosomal dyes in SCLC cells overexpressing DCP2 following chemotherapeutic drug treatment. Scale bar represents 25 μm. **G-H** Mtphagy images show mitophagy fluorescence, and merged images show colocalization between mitochondrial and lysosomal dyes in DCP2-knockdown SCLC cells after chemotherapeutic drug treatment. Scale bar represents 25 μm. **I-J** Mtphagy images show mitophagy fluorescence, and merged images show colocalization between mitochondrial and lysosomal dyes in SCLC cells with simultaneous knockdown of METTL3 and DCP2 following chemotherapeutic drug exposure. Scale bar represents 25 μm

To determine whether regulation of DCP2 levels by METTL3 contributes to SCLC chemoresistance, we constructed DCP2-overexpressing and DCP2-knockdown cells in chemoresistant and chemosensitive cell lines, respectively (Fig. S3A-B). The IC50 of chemotherapeutic drugs, including CDDP and VP16, was determined following 24 hours of treatment. Knockdown of DCP2 in chemosensitive cells significantly increased the IC50 values of chemotherapeutic drugs (Fig. 5A), while overexpression of DCP2 in chemoresistant cells significantly decreased the IC50 values (Fig. 5B). Further rescue experiments showed that when METTL3 was knocked down, the sensitivity of cells to chemotherapeutic drugs was increased, and the IC50 values decreased.However, when both METTL3 and DCP2 were knocked down simultaneously, chemoresistance caused by METTL3 was restored (Fig. 5C-D). We also analysed DCP2 expression in subcutaneously transplanted nude mouse tumours in which we knocked down or overexpressed METTL3. Again, we found that DCP2 expression decreased with increasing METTL3 expression (Fig. S3C). These results suggest that DCP2 is involved in the METTL3-mediated chemoresistance of SCLC.

To further explore possible downstream mechanisms by which METTL3 mediates chemoresistance, we performed GO pathway enrichment analysis of the MeRIP-seq data. GO analysis showed that autophagy-related pathways were highly enriched, and among the 12 autophagy-related pathways with significant differences, five pathways belonged to the “mitochondrial autophagy with selective autophagy” category (Fig. 5E). To confirm whether mitochondrial autophagy is involved in SCLC chemotherapy resistance, confocal microscopy was used to evaluate mitochondrial autophagy in chemosensitive and drug-resistant cells. When the same concentration of chemotherapeutic drugs was used to induce mitochondrial autophagy in chemosensitive and chemoresistant cells, the Mtphagy fluorescence intensity, representing mitochondrial autophagy, was significantly higher in chemoresistant cells than in chemosensitive cells (Fig. S3D). However, after overexpression of DCP2 in chemoresistant cells, Mtphagy fluorescence intensity was significantly decreased, and this finding was consistent across the two cell lines analysed (Figs. 5F and S3E-F). After knockdown of DCP2 in chemosensitive cells, Mtphagy fluorescence intensity was significantly increased (Fig. 5G-H and S3G-H). Further experiments showed that when METTL3 was knocked down in chemoresistant cell lines, decreased mitochondrial autophagy was observed, and this decreased mitochondrial autophagy was rescued by simultaneous knockdown of METTL3 and DCP2 (Figs. 5I-J and S3I-J). To summarize, we found that DCP2, a METTL3 target gene, was involved in the chemoresistance process of SCLC by affecting the level of mitophagy.

### DCP2 regulates Pink1-Parkin pathway-mediated mitophagy

**Fig. 6:**
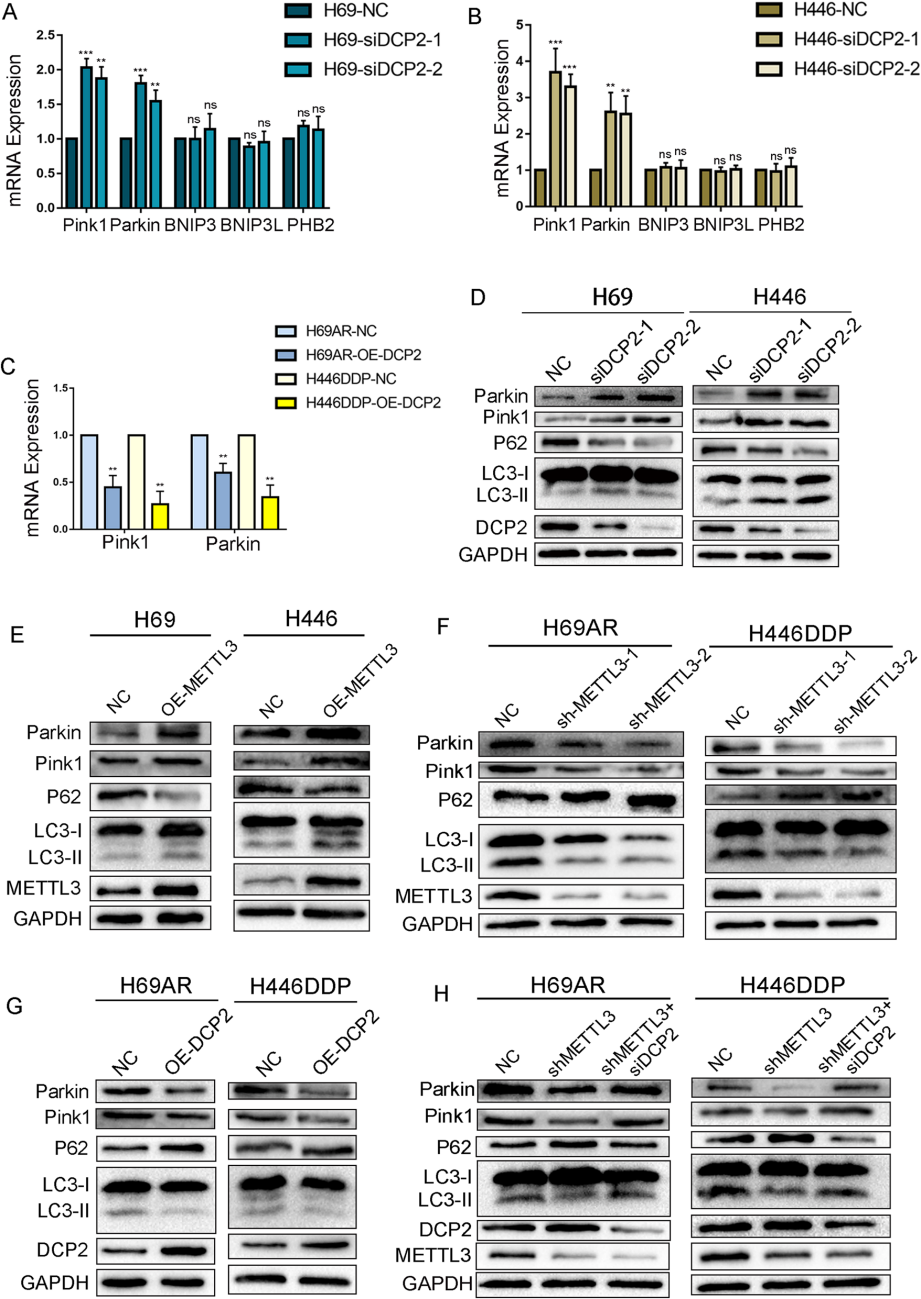
DCP2 regulates mitophagy mediated by the Pink1-Parkin pathway. **A-B** qRT–PCR analysis of the mRNA expression of mitophagy-related proteins after knockdown of DCP2 in the drug-sensitive cell lines H69 and H446. **P < 0.01; ***P < 0.001; ns, not significant. **C** qRT–PCR analysis of the mRNA expression of mitophagy-related proteins after overexpression of DCP2 in the drug-resistant cell lines H69AR and H446DDP. **P < 0.01. **D-E** Western blot analysis of mitophagy-related protein expression after knockdown of DCP2 (**D**) and overexpression of METTL3 (**E**) in the drug-sensitive cell lines H69 and H446. **F-G** Western blot analysis of mitophagy-related protein expression after knockdown of METTL3 (**F**) and overexpression of DCP2 (**G**) in the drug-resistant cell lines H69AR and H446DDP. **H** Western blot analysis of mitophagy-related protein expression after simultaneous METTL3 and DCP2 knockdown in the drug-resistant cell lines H69AR and H446DDP

In previous studies, it has been stated that mitochondrial autophagy occurs through two classical pathways: the Pink1-Parkin-mediated ubiquitin-dependent pathway, which is triggered by membrane depolarization, and receptor-mediated pathways, the core components of which include BNIP3 (BCL2-interacting protein 3), BNIP3L/NIX (BCL2-interacting protein 3-like), and PHB2 [35, 36]. To explore whether DCP2 regulates mitochondrial autophagy in SCLC through either of these known pathways, changes in the expression of mitophagy genes were analysed in DCP2 knockdown chemosensitive cells. DCP2 knockdown resulted in increased Pink1 and Parkin mRNA levels, whereas there were no significant changes in the mRNA levels of BNIP3, BNIP3L or PHB2 (Fig. 6A-B). Likewise, Pink1 and Parkin mRNA levels decreased in chemoresistant cells overexpressing DCP2 (Fig. 6C). 7-Methylguanosine diphosphate (m7GDP) is a drug that has been shown to inhibit the activity of Decapping Scavenger (DcpS) [37, 38]. Chemosensitive cells were treated with varying concentrations of m7GDP, and Pink1 and Parkin mRNA levels were analysed. We found that Pink1 and Parkin expression changed with increasing m7GDP concentrations (Fig. S4A-B). These results indicate that the mRNA stability of Pink1 and Parkin is affected by DCP2 and that DCP2 can induce the degradation of Pink1 and Parkin.

To further characterize changes in mitochondrial autophagy in SCLC cells, we analysed the protein levels of Pink1, Parkin, LC3, and P62. Compared to chemosensitive cells, chemoresistant cells showed significantly increased expression levels of mitochondrial autophagy proteins (Fig. S4C), which was consistent with the results of the mitochondrial autophagy fluorescence experiments. When DCP2 was knocked down or METTL3 was overexpressed in chemosensitive cells, the expression levels of mitochondrial autophagy proteins were significantly increased (Fig. 6D-E). Moreover, when DCP2 was overexpressed or METTL3 was knocked down in chemoresistant cells, mitochondrial autophagy protein levels were decreased (Fig. 6F-G). When we extracted the mitochondria and detected the expression of Parkin and Pink1 protein in them separately, we obtained the same conclusion as in the whole cell lysate (Fig. S4D-G). To further characterize mitochondrial autophagy, we also detected the important autophagic proteins Beclin 1 and Vps34. The results showed that overexpression of METTL3 or knockdown of DCP2 in chemotherapy-sensitive SCLC cells increased the expression levels of Beclin 1 and Vps34, while knockdown of METTL3 or overexpression of DCP2 in chemotherapy-resistant SCLC cells significantly decreased the expression levels of Beclin 1 and Vps34 (Fig. S5A-D).

Further rescue experiments showed that when METTL3 and DCP2 were knocked down simultaneously in chemoresistant cells, the changes in autophagy protein levels caused by METTL3 knockdown were restored (Fig. 6H). These results suggest that METTL3 regulation of DCP2 levels contributes to SCLC chemotherapy resistance by regulating Pink1-Parkin-mediated mitochondrial autophagy.

### DCP2 regulates mitochondrial damage in SCLC

**Fig. 7:**
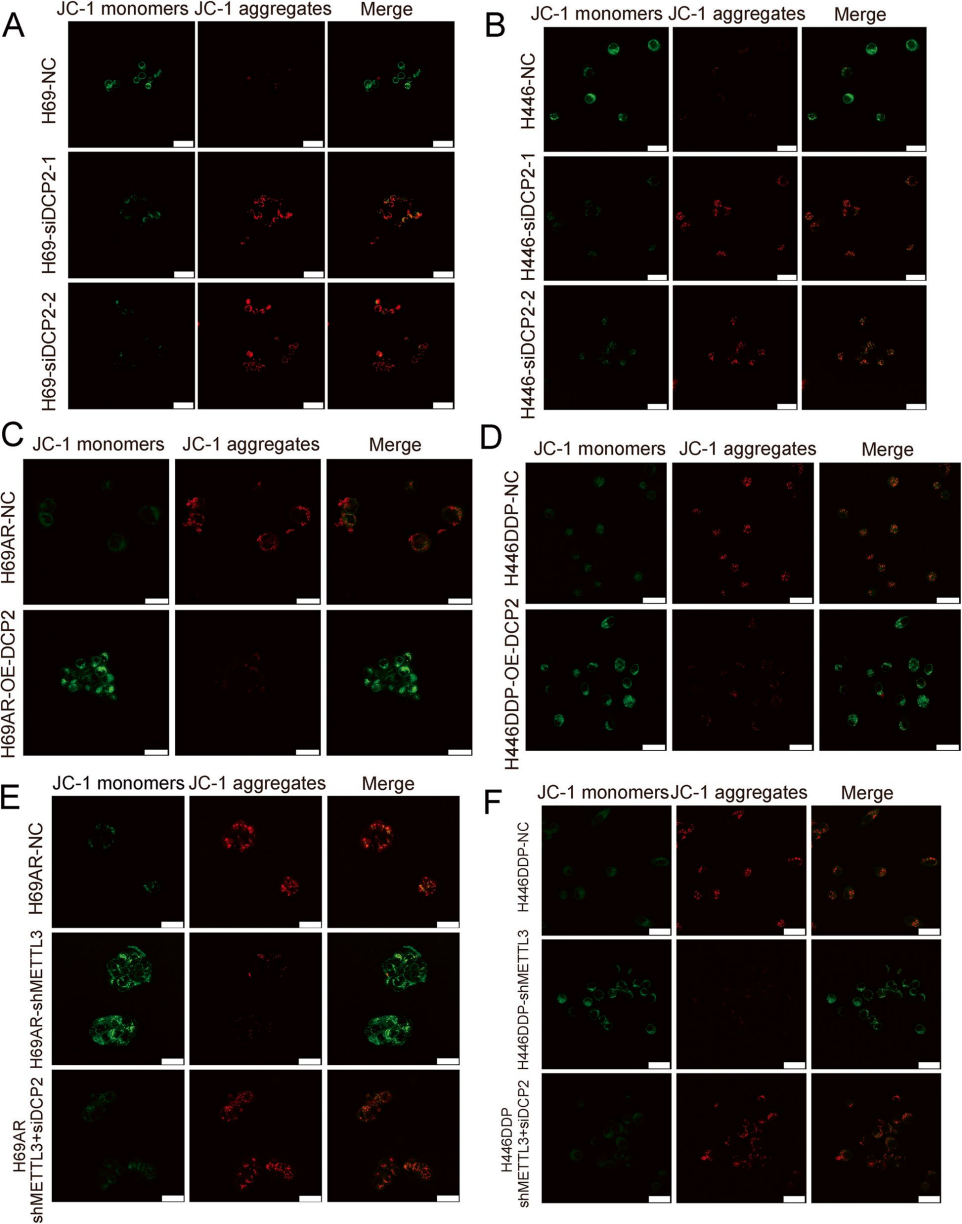
DCP2 regulates mitochondrial damage levels in SCLC cells. **A-B** The mitochondrial membrane potential detection kit (JC-1) was used to detect differences in mitochondrial damage levels in DCP2 knockdown SCLC cells following treatment with the chemotherapeutic drugs CDDP and VP16 (when the mitochondrial membrane potential is high, JC-1 aggregates in the mitochondrial matrix to form polymers and produces red fluorescence; when the mitochondrial membrane potential is low, JC-1 exists in monomer form and cannot aggregate in the mitochondria in the matrix, which produces green fluorescence). **C-D** A mitochondrial membrane potential detection kit (JC-1) was used to detect differences in mitochondrial damage levels in DCP2-overexpressing SCLC cells following treatment with chemotherapeutic drugs. **E-F** A mitochondrial membrane potential detection kit (JC-1) was used to detect differences in mitochondrial damage levels in SCLC cells with simultaneous knockdown of METTL3 and DCP2 following treatment with chemotherapeutic drugs

Numerous studies have confirmed that chemoresistance is related to mitochondrial dysfunction. Mitochondrial autophagy is the targeted phagocytosis and destruction of mitochondria by autophagy and is considered the main mechanism of mitochondrial quality control [39, 40]. Therefore, we speculated that there were changes in the level of mitochondrial damage during SCLC chemotherapy resistance. Mitochondrial membrane potential (MMP) and mitochondrial reactive oxygen species (mtROS) are key indicators of mitochondrial activity because they reflect the processes of electron transport and oxidative phosphorylation, which are the driving forces behind ATP production in both biological processes and human cancer pathogenesis [41]. We found that knockdown of DCP2 in SCLC chemotherapy-sensitive cells significantly decreased the production of mitochondrial ROS (Fig. S6A) and significantly increased MMP (Fig. 7A-B), indicating that knockdown of DCP2 can reduce mitochondrial damage induced by chemotherapy drugs. Similarly, after DCP2 overexpression in chemotherapy-resistant cells, the DCP2 overexpression group had increased mitochondrial damage induced by chemotherapy drugs (mtROS fluorescence intensity increased, MMP fluorescence intensity decreased) (Figs. 7C-D and S6B). Further rescue experiments showed that when METTL3 and DCP2 were knocked down simultaneously in chemoresistant cells, the mitochondrial damage induced by chemotherapy drugs caused by METTL3 was restored (Figs. 7E-F and S6C). In conclusion, regulation of DCP2 by METTL3 in SCLC alters the level of mitochondrial damage induced by chemotherapeutic drugs.

### METTL3 inhibitor STM2457 reverses chemoresistance of SCLC cells in vitro and in vivo

**Fig. 8:**
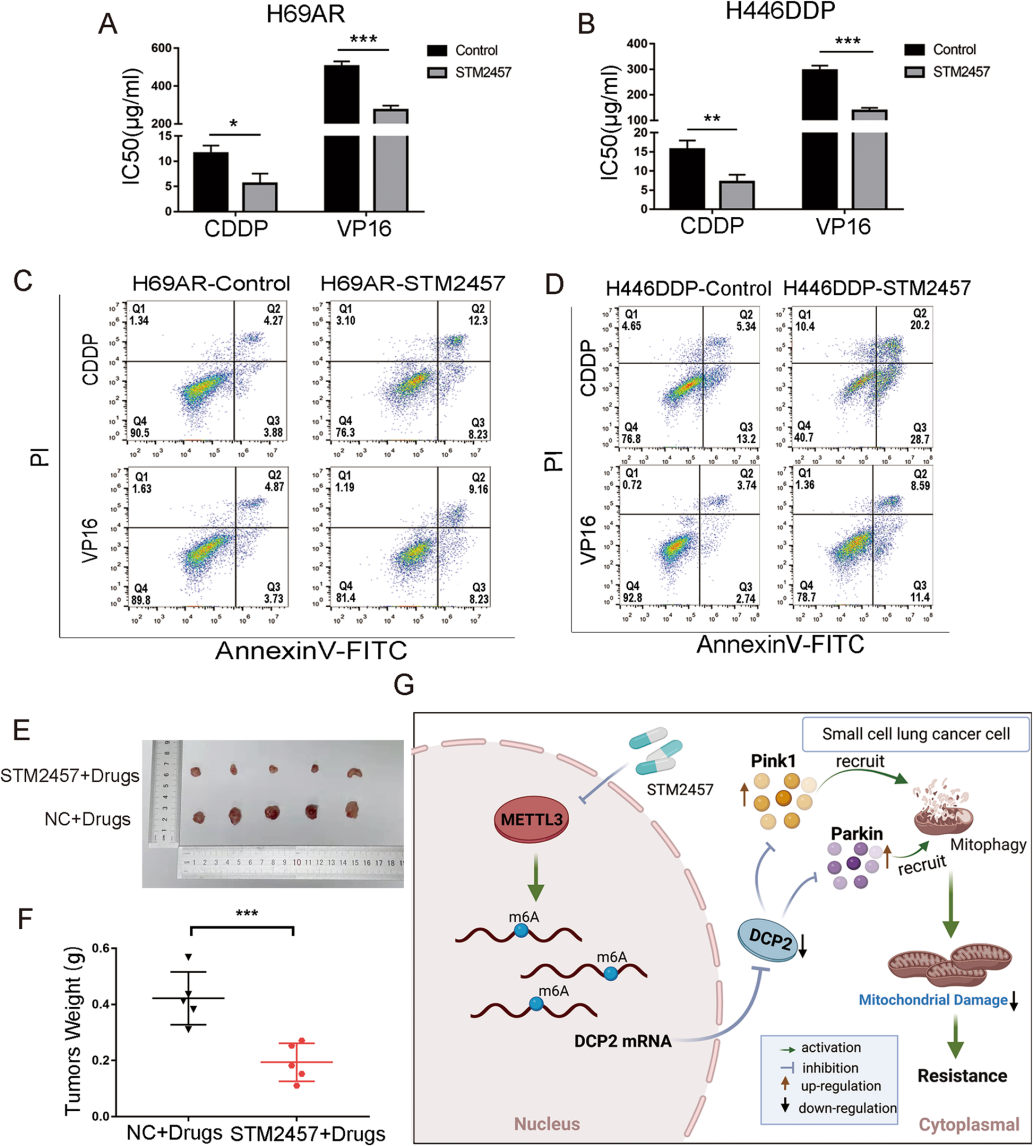
The METTL3 inhibitor STM2457 reverses chemoresistance in SCLC cells in vitro and in vivo. **A-B** CCK-8 assay shows that STM2457 (6.25 μM) decreased the IC50 values of chemotherapeutic agents for chemoresistant H69AR and H446DDP cells. *P < 0.05; **P < 0.01; ***P < 0.001. **C-D** Cell apoptosis and arrest were evaluated by flow cytometry in STM2457-treated (6.25 μM) SCLC cells after chemotherapeutic drug treatment. **E-F** Effect of STM2457 (6.25 μM) on resistance to chemotherapeutic drugs in nude mice with implanted tumours and measurement of tumour weights for different treatment groups. ***P < 0.001. **G** Mechanism diagram: The m6A methyltransferase METTL3 promotes SCLC chemoresistance by targeting DCP2, which regulates mitophagy and mitochondrial damage via the Pink1-Parkin pathway

STM2457 is a novel, highly selective, orally active METTL3 inhibitor, that has been previously reported in a study of acute leukemia [42]. To determine a suitable concentration of STM2457 that can inhibit METTL3 expression in SCLC cells without affecting the proliferation of normal lung epithelial cells, we treated normal lung epithelial cells(HBE) with different concentrations of STM2457. When the concentration of STM2457 was 6.25 μM, there was no effect on the proliferation of normal HBE lung epithelial cells (Fig. S7A). Western blotting showed that treatment with this concentration of STM2457 increased the expression levels of DCP2 and decreased the expression levels of Pink1 and Parkin in H69AR and H446DDP cells (Fig. S7B). After 24 hours of treatment with CDDP or VP16, we determined the IC50 and cell apoptosis. The IC50 values of CDDP and VP16 were significantly decreased after treatment with STM2457 (Fig. 8A-B). The flow cytometry results also showed that the proportion of apoptotic cells was higher in the STM2457 treatment group than the control group (Figs. 8C-D and S7C-D).

To observe whether STM2457 affected SCLC drug resistance in vivo, H69AR cells treated with 6.25 μM STM2457 for 24 hours were subcutaneously injected into nude mice. When combined with chemotherapy drugs, STM2457 significantly inhibited the growth rate of the xenografts (Fig. S7E) and significantly reduced the volume of xenografts after chemotherapy (Fig. 8E-F). In conclusion, the METTL3 inhibitor STM2457 could reverse SCLC chemoresistance and has the potential to treat chemoresistance in SCLC patients.

## Discussion

The mechanisms of chemotherapy resistance in malignant tumours are very complex, and most result in multidrug resistance [26, 43]. m6A methylation is an epigenetic modification of RNA that mainly affects mRNA stability, nuclear output, and translation [44]. Many studies have shown that m6A plays an important role in various biological processes and is especially important in drug resistance [45].

Few studies have examined the role of the m6A modification in SCLC chemotherapy resistance. The present study demonstrated for the first time that METTL3, an important m6A methyltransferase, induces SCLC chemoresistance. By transcriptome sequencing analysis and in vivo and in vitro experiments, we found that METTL3 was highly expressed in chemoresistant cells and promoted chemoresistance. METTL3 interacts with METTL14 in the heterodimer formed, which deposits m6A on mRNAs [46]. Previous studies suggested that METTL14 was an inactive methyltransferase for the disruption of its active site motif [47]. Biochemical and structural evidence also indicated that METTL14 has no detectable activity [34, 48, 49]. METTL14 primarily serves as a platform for substrate interaction in the binary complex. When METTL3 and METTL14 form a heterodimeric complex, the catalytic activity of METTL3 is dramatically stimulated [48]. in our study, the heatmap of mRNA expression based on whole-transcriptome sequencing revealed the high relative abundance of METTL14 in chemotherapy-sensitive and chemotherapy-resistant cells, and METTL14 expression was not significantly different in SCLC. This result implied the significant effect of METTL3 with differential expression on methyltransferase activity in SCLC.

Transcriptome sequencing analysis of surgical specimens from local SCLC patients also showed that METTL3 expression was significantly associated with poor prognosis. Previous studies have confirmed that METTL3-mediated m6A methylation is associated with chemotherapy resistance in various solid tumours. For example, in non-small cell lung cancer, METTL3-mediated m6A methylation increased the stability of YAP mRNA by regulating Malat1-miR-1914-3p-Yap axis-induced chemotherapy resistance [50]. In breast cancer and colorectal cancer, METTL3-mediated m6A methylation has been repeatedly linked to doxorubicin and oxaliplatin resistance [51, 52]. In this study, we observed that differential expression of METTL3 expression influenced the IC50 of CDDP and VP16 treatment in SCLC cells, and regulating METTL3 expression alone cannot completely reverse the IC50 of chemically resistant cells to the same level as that of chemically sensitive parent cells, which may be related to the unclear mechanism of SCLC chemotherapy resistance and the complex process of SCLC biological behaviour regulated by multiple factors. Notably, it was reported that platinum drugs could increase the overall cellular m6A level via the SNHG3/miR-186-5p/METTL3 axis, and that it was involved in platinum resistance [53]. This finding may be explained by the fact that, compared to VP16 susceptibility, CDDP susceptibility seems to be more dependent on METTL3 expression.

To explore possible downstream mechanisms by which METTL3 promotes SCLC chemoresistance, we applied RNA-seq and MeRIP-Seq to jointly identify downstream targets of METTL3-mediated m6A methylation. Based on the comprehensive analysis of sequencing data and in vitro experiments, we determined that DCP2 m6A methylation levels in SCLC were regulated by METTL3, and highly m6A-modified DCP2 mRNA was degraded, resulting in decreased DCP2 protein expression. Transcripts with high m6A levels may experience aggregation and degradation, depending on subsequent recognition by m6A readers. Degradation after m6A methylation has been confirmed in many studies, and it primarily involves selective recognition by the m6A reading protein YTH domain family 2 (YTHDF2). The carboxy-terminal domain of YTHDF2 selectively binds to m6A-containing mRNA, while the N-terminal domain is responsible for localizing the YTHDF2-mRNA complex to sites of cellular RNA decay to regulate mRNA degradation [54, 55]. DCP2 is a major decapping enzyme during 5′ to 3′ mRNA decay, and the 5′ mRNA cap determines nuclear and cytoplasmic fate by interacting with a number of specialized cap-binding proteins that mediate important cellular functions, including pre-mRNA processing, nuclear export, translation initiation, and bulk mRNA decay [13, 15, 56]. There are no prior reports on whether DCP2 is involved in tumour drug resistance. In the present study, we found that DCP2, a METTL3 target gene, negatively regulates SCLC chemotherapy resistance. This finding has important implications for the future development of drugs that can reverse SCLC chemoresistance.

To further characterize the molecular mechanism by which DCP2 negatively regulates SCLC chemotherapy resistance, we performed GO pathway enrichment analysis on our m6A-seq data and found that the mitochondrial autophagy pathway was highly enriched. Furthermore, by analysing mitophagy fluorescence and the expression of autophagy-related proteins, we found that DCP2 regulates mitophagy and mitochondrial damage levels by controlling Pink1 and Parkin expression. Mitochondrial autophagy is a selective autophagy process that aims to eliminate dysfunctional or nonessential mitochondria to maintain cell homeostasis [22]. The Pink1-Parkin pathway is a classic pathway for the activation of mitochondrial autophagy. When mitochondria are damaged and depolarized, Pink1 accumulates on the outer membrane and recruits Parkin. Pink1 activates Parkin by phosphorylating Parkin on Thr175, Thr217 and Ser65, thus initiating mitochondrial autophagy [57]. Our results indicate that DCP2 can regulate the expression of Pink1 and Parkin, which connects METTL3-mediated m6A methylation and mitochondrial autophagy. Mitochondrial autophagy has been repeatedly shown to play a role in chemoresistance in various cancer types, including adriamycin resistance in colorectal cancer and cisplatin resistance in non-small cell lung cancer [58, 59]. However, there are few reports on the role of mitochondrial autophagy in SCLC chemotherapy resistance. In the present study, we found that mitochondrial autophagy promoted SCLC chemotherapy resistance. Mitochondrial autophagy was increased in chemotherapy-resistant cells compared to chemosensitive cells, and the level of mitochondrial autophagy was altered by changes in METTL3 and DCP2 expression.

Mechanistically, in chemoresistant cells, increased METTL3 expression led to increased m6A methylation of DCP2 transcripts, and transcripts with high m6A methylation levels were degraded, resulting in decreased DCP2 mRNA and protein levels. The decreased DCP2 expression further regulated Pink1 and Parkin mRNA stability, increasing mitophagy and decreasing mitochondrial damage. Ultimately, these events contribute to SCLC chemotherapeutic drug resistance (Fig. 8G). These findings suggest that METTL3 could become a promising clinical target for reversing SCLC chemoresistance.

Finally, we examined the effect of a novel METTL3 inhibitor, STM2457, on SCLC chemoresistance. STM2457 can inhibit the catalytic activity of METTL3 and has been previously shown to affect the proliferation of leukaemia and bile duct tumour cells [42, 54]. In the present study, STM2457 significantly changed the sensitivity of SCLC cells to chemotherapeutic drugs in vitro and in vivo and reversed SCLC chemoresistance. These findings provide useful insight for the development of clinical antitumour drugs and the future application of STM2457 to overcome SCLC drug resistance.

## Conclusions

Our study confirmed that the m6A methyltransferase METTL3 participates in the chemoresistance process of SCLC by promoting m6A of DCP2, inducing Pink1-Parkin pathway mediated mitophagy and reducing mitochondrial damage. At the same time, the new METTL3 inhibitor STM2457 has also been proved to have the effect of regulating the chemotherapy resistance of SCLC. Therefore, METTL3 is a promising new therapeutic target for reversing chemotherapy resistance in SCLC.

## Supplementary Information

**Additional file 1.**

## Data Availability

The data sets generated during and/or analyzed during the current study are available from the corresponding author on reasonable request.

## Abbreviations

SCLC: Small cell lung cancer
GEO: Gene Expression Omnibus
IC50: Half maximal inhibitory concentration
OS: Overall survival
GO: Gene Ontology
METTL3: Methyltransferase-like 3
m6A: N6-methyladenosine
DCP2: Decapping Protein 2
m7GDP: 7-methylguanosine diphosphate
CCK8: Cell counting kit-8
CDDP: Cisplatin
VP16: Etoposide
MeRIP-seq: Methylated RNA immunoprecision sequencing
DcpS: Decapping Scavenger
