# Supplementary material for: METTL3 promotes chemoresistance in small cell lung cancer by inducing mitophagy
Source: J Exp Clin Cancer Res. 2023 Mar 17;42:65. doi: 10.1186/s13046-023-02638-9 (PMC10022264; doi:10.1186/s13046-023-02638-9)
Supplement: Supplementary file 1 — Additional file 1. [file 13046_2023_2638_MOESM1_ESM.docx]

**Supplement Figure Legends**

**Table S1：Clinical information of local data**

| **ID** | **Age** | **Sex** | **Smoking** | **T** | **N** | **M** | **Stage** | **Status** | **days** | **Surgery** | **Post-operative treatment** |
| --- | --- | --- | --- | --- | --- | --- | --- | --- | --- | --- | --- |
| P428785 | 45 | Male | Yes | 2b | 2 | 0 | III | alive | 1665 | Yes | Chemotherapy (IEP) |
| P433364 | 60 | Male | Yes | 1b | 0 | 0 | I | death | 3027 | Yes | Chemotherapy (EP) |
| P434465 | 63 | Male | Yes | 3 | 0 | 0 | II | death | 2217 | Yes | Chemotherapy (TP) |
| P439398 | 28 | Female | No | 1b | 0 | 0 | I | death | 2951 | Yes | Chemotherapy  (Unknown) |
| P440680 | 52 | Male | Yes | 2 | 2 | 0 | III | death | 3273 | Yes | Chemotherapy (EP) |
| P441448 | 54 | Male | Yes | 3 | 2 | 0 | III | death | 2886 | Yes | Chemotherapy (EP) |
| P445971 | 50 | Male | No | 1b | 2 | 0 | III | alive | 656 | Yes | Chemotherapy (EP) |
| P450286 | 74 | Male | No | 1b | 0 | 0 | I | death | 251 | Yes | Chemotherapy (EP) |
| P460095 | 62 | Female | No | 2 | 2 | 0 | III | alive | 365 | Yes | Unknown |
| P461632 | 74 | Male | Yes | 2b | 3 | 0 | III | alive | 6 | Yes | Unknown |
| P462236 | 61 | Female | Yes | 2 | 0 | 0 | I | alive | 789 | Yes | Unknown |
| P462696 | 64 | Male | Yes | 3 | 2 | 0 | III | alive | 495 | Yes | Chemotherapy (EP) |
| P466839 | 52 | Male | Yes | 2b | 2 | 0 | III | alive | 356 | Yes | Unknown |
| P468720 | 56 | Male | Yes | 2a | 2 | 0 | III | alive | 612 | Yes | Chemotherapy (EP) |
| P470298 | 69 | Female | Yes | 2b | 2 | 0 | III | alive | 443 | Yes | Chemotherapy  (Unknown) |
| P472257 | 76 | Male | Yes | 2a | 0 | 0 | II | alive | 317 | Yes | Unknown |
| P475716 | 58 | Male | Yes | 2a | 2 | 0 | III | alive | 1055 | Yes | Unknown |
| P476001 | 70 | Male | Yes | 1a | 0 | 0 | I | alive | 1405 | Yes | Chemotherapy (EP) |
| P476512 | 62 | Male | Yes | 2a | 2 | 0 | III | death | 2724 | Yes | Unknown |
| P481433 | 71 | Male | Yes | 1 | 2 | 0 | III | alive | 382 | Yes | Unknown |
| P496235 | 70 | Male | Yes | 3 | 2 | 0 | III | alive | 320 | Yes | Unknown |
| P501599 | 53 | Male | Yes | 2a | 1 | 0 | II | alive | 1281 | Yes | Chemotherapy (EP) |
| P502262 | 75 | Male | Yes | 2 | 2 | 1 | IV | death | 801 | Yes | Unknown |
| P503321 | 63 | Male | Yes | 2a | 1 | 0 | II | alive | 1566 | Yes | Chemotherapy (EP) |
| P503325 | 60 | Male | Yes | 1a | 0 | 0 | I | death | 287 | Yes | Unknown |
| P510123 | 59 | Female | No | 3 | 2 | 0 | III | alive | 521 | Yes | Chemotherapy (EP) |
| P511915 | 70 | Male | Yes | 2a | 2 | 0 | III | death | 2083 | Yes | Chemotherapy (EP) |
| P526443 | 59 | Male | Yes | 2a | 0 | 0 | I | alive | 1044 | Yes | Unknown |
| P527622 | 50 | Female | No | 2a | 0 | 0 | I | death | 1882 | Yes | Chemotherapy (EP) |
| P528065 | 50 | Male | Yes | 3 | 0 | 1b | IV | alive | 263 | Yes | Chemotherapy (EP) |
| P529039 | 64 | Male | Yes | 1b | 0 | 0 | I | alive | 313 | Yes | Unknown |
| P530825 | 65 | Male | Yes | 2a | 2 | 0 | III | alive | 295 | Yes | Chemotherapy (EP) |
| P534673 | 50 | Male | Yes | 2b | 2 | 0 | III | alive | 395 | Yes | Chemotherapy (EP) |
| P538110 | 23 | Male | Yes | 2 | 2 | 0 | III | death | 1561 | Yes | Chemotherapy (EP) |
| P541687 | 66 | Female | No | 2a | 0 | 0 | I | alive | 821 | Yes | Unknown |
| P547393 | 64 | Male | Yes | 2a | 0 | 0 | I | alive | 331 | Yes | Chemotherapy (EP) |
| P558674 | 56 | Male | Yes | 2a | 0 | 0 | I | death | 1683 | Yes | Chemotherapy (EP) |
| P573341 | 56 | Male | Yes | 2a | 0 | 0 | I | death | 1524 | Yes | Unknown |
| P573885 | 75 | Male | Yes | 2a | 0 | 0 | II | death | 1298 | Yes | Radiotherapy |
| P581639 | 65 | Male | Yes | 1b | 2 | 0 | III | death | 1150 | Yes | Chemotherapy (EP) |
| P590387 | 65 | Male | Yes | 1b | 0 | 0 | I | death | 1297 | Yes | Chemotherapy (EP) |
| P594699 | 74 | Male | No | 1b | 1 | 0 | II | death | 872 | Yes | Unknown |
| P596385 | 66 | Female | No | 2a | 2 | 0 | III | death | 1291 | Yes | Chemotherapy (EP) |
| P601078 | 53 | Female | No | 1b | 2 | 0 | III | death | 1242 | Yes | Unknown |
| P602449 | 62 | Male | Yes | 1a | 0 | 0 | I | death | 991 | Yes | Chemotherapy  (Unknown) |
| P610023 | 68 | Male | Yes | 2b | 0 | 0 | II | death | 1160 | Yes | Chemotherapy (EP) |
| P617679 | 58 | Male | Yes | 2a | 0 | 0 | I | death | 994 | Yes | Chemotherapy (EP) |
| P625511 | 60 | Male | Yes | 1a | 0 | 0 | I | death | 1013 | Yes | Chemotherapy (TC) |
| P643252 | 56 | Male | Yes | 2a | 2 | 0 | III | death | 773 | Yes | Chemotherapy (EP) |
| P649680 | 55 | Male | Yes | 2a | 0 | 0 | I | death | 86 | Yes | Chemotherapy (EP) |
| P656602 | 58 | Male | Yes | 1c | 1 | 0 | II | death | 753 | Yes | Unknown |
| P664781 | 72 | Male | Yes | 1 | 2 | 0 | III | alive | 362 | Yes | Chemotherapy  (Unknown) |
| P673233 | 77 | Male | Yes | 3 | 0 | 0 | II | death | 627 | Yes | Chemotherapy (EP) |
| P685209 | 64 | Male | NA | 3 | 0 | 0 | II | death | 551 | Yes | Unknown |
| P705910 | 64 | Male | Yes | 3 | 2 | 0 | III | death | 382 | Yes | Chemotherapy (EP) |
| P715285 | 63 | Male | Yes | 4 | 0 | 0 | III | death | 295 | Yes | Radiotherapy |
| P724807 | 49 | Female | No | 4 | 0 | 0 | III | death | 240 | Yes | Chemotherapy (EP) |
| P735897 | 73 | Male | No | 1 | 2 | 0 | III | death | 29 | Yes | Unknown |

Abbreviations: IEP: Isocyclophosphamide, Etoposide and Cisplatin; EP: Etoposide and Cisplatin; TC: Taxol and Carboplatin; TP: Taxol and Cisplatin.

**Table S2. Primers for real-time PCR**

| mRNA | Forward primer (5'-3') | Reverse primer (5'-3') |
| --- | --- | --- |
| METTL3 | AGCCTTCTGAACCAACAGTCC | CCGACCTCGAGAGCGAAAT |
| DCP2 | TGAAGACCAGTTGCTAGAACATGC | \| GTCCAAGATTTTCATTATAGCATTATGGT \| \| --- \| |
| DCP2-m6A | AATGCTATAATGAAAATCTTGGACC | CACCCACTCAAATTCAACAGG |
| KPNB1 | CCACTTTCCTTGTGGAACTGT | CTCTGCTGATATTGTGCCTTGA |
| DAAM1 | GGTGGACGAGGTATTTCATTCAT | TCGCAGCCGATACGTGATTTC |
| Pink1 | GCCTCATCGAGGAAAAACAGG | GTCTCGTGTCCAACGGGTC |
| Parkin | GTGTTTGTCAGGTTCAACTCCA | AAAATCACACGCAACTGGTC |
| BNIP3 | CAGGGCTCCTGGGTAGAACT | CTACTCCGTCCAGACTCATGC |
| BNIP3L | ATGTCGTCCCACCTAGTCGAG | TGAGGATGGTACGTGTTCCAG |
| PHB2 | GTGCGCGAATCTGTGTTCAC | GATAATGGGGTACTGGAACCAAG |
| GAPDH | CAATGACCCCTTCATTGACC | GACAAGCTTCCCGTTCTCAG |

**Table S3. Antibody Information**

| **Antibodies** | SOURCE | Catalog Number |
| --- | --- | --- |
| METTL3 | Abcam | Cat#: ab195352 |
| PARP | Cell Signaling Technology | Cat#: 9532 |
| BCL-2 | Proteintech | Cat#:60178-1-ig |
| BAX | Proteintech | Cat#:50599-2-Ig |
| GAPDH | Bioworld | Cat#: ap0063 |
| DCP2 | Abcam | Cat#: ab127185 |
| Pink1 | Proteintech | Cat#: 23274-1-ap |
| Parkin | Proteintech | Cat#:14060-1-ap |
| P62 | Cell Signaling Technology | Cat#: 88588 |
| LC3 | Cell Signaling Technology | Cat#: 83506 |
| Goat Anti-Rabbit IgG  antibody (HRP) | Abcam | Cat#: ab205718 |
| Goat Anti-Mouse IgG  antibody (HRP) | Abcam | Cat#: ab97240 |

**Table S4. Sequences of shRNA**

| shRNA | Target Seq |
| --- | --- |
| shMETTL3-1 | gcAAGTATGTTCACTATGAAA |
| shMETTL3-2 | cgTCAGTATCTTGGGCAAGTT |

**Table S5. Sequences of siRNA**

| siRNA | sense（5'-3'） | antisense（5'-3'） |
| --- | --- | --- |
| siDCP2-1 | CCACGGAAACUUCAGGAUATT | UAUCCUGAAGUUUCCGUGGTT |
| siDCP2-2 | GCAUGUAAUGGACAUUGCATT | \| UGCAAUGUCCAUUACAUGCTT \| \| --- \| |

**
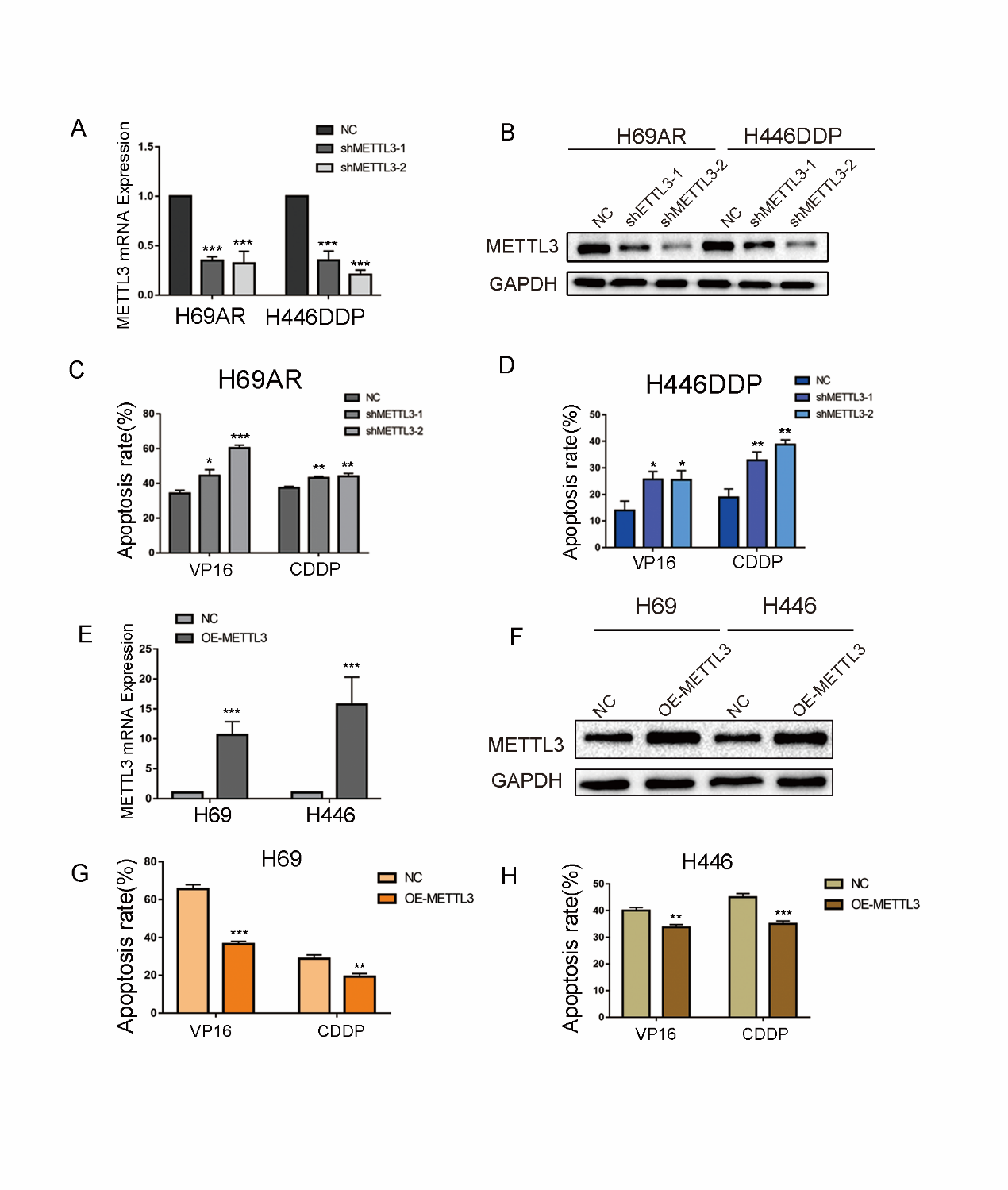
**

**Figure S1. METTL3 induces chemoresistance in SCLC cells**. **(A-B)** Inhibition of METTL3 expression by transfection of METTL3 shRNA in H69AR and H446DDP cells. ***P < 0.001. **(C-D)** Bar graph showing that knockdown of METTL3 significantly increased the proportion of apoptotic cells in chemotherapy-resistant cell lines. *P < 0.05, **P < 0.01; ***P < 0.001. **(E-F)** Overexpression of METTL3 using lentivirus in H69 and H446 cells. ***P < 0.001. **(G-H)** Bar graph showing that overexpression of METTL3 significantly reduced the proportion of apoptotic cells among chemotherapy-sensitive cells. **P < 0.01; ***P < 0.001
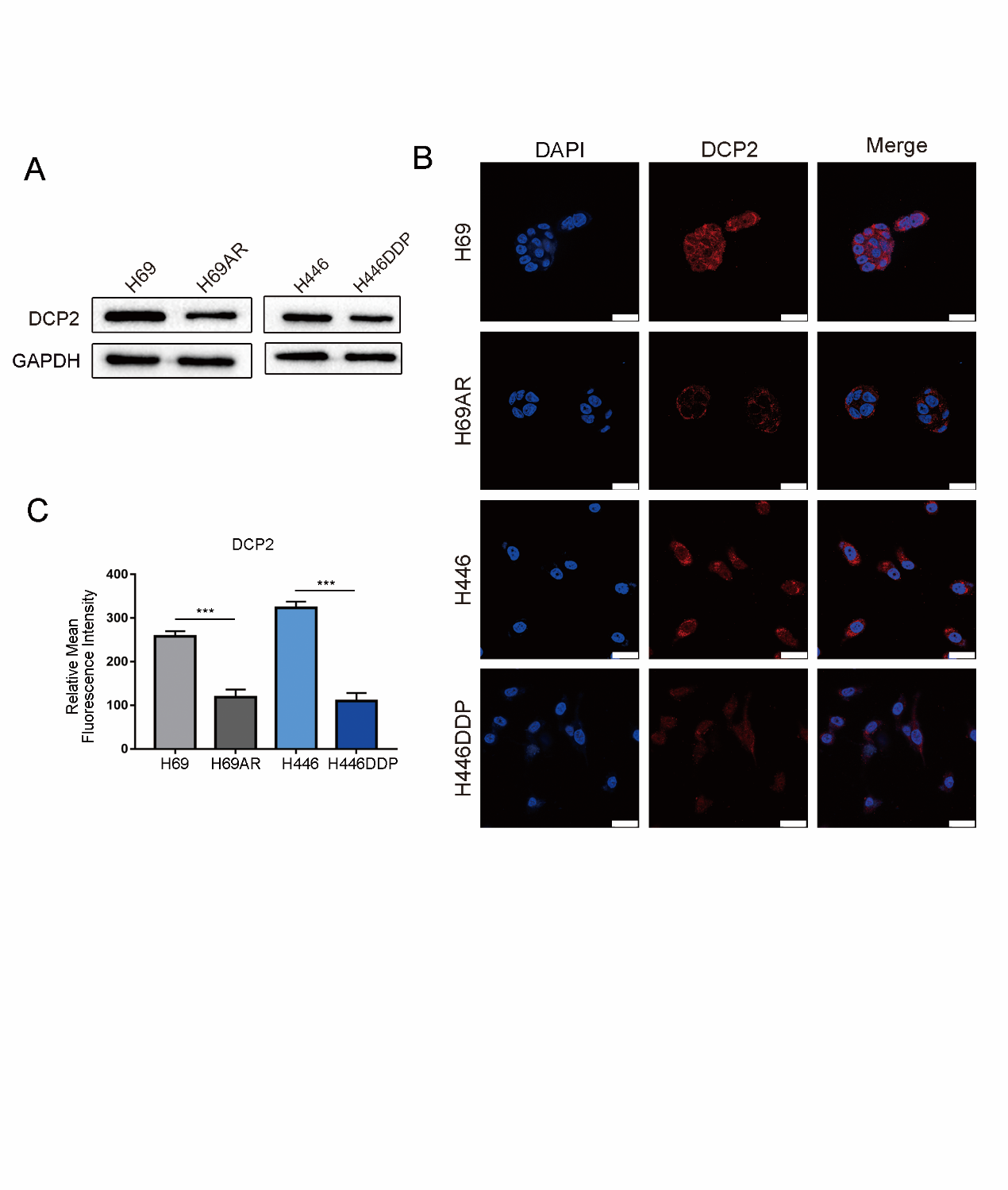


**Figure S2. DCP2 is highly expressed in chemosensitive cells. (A)** Western blot analysis of DCP2 expression in two pairs of chemosensitive and chemoresistant SCLC cells, H69/H69AR and H446/H446DDP. **(B-C)** Immunofluorescence analysis of DCP2 expression in H69/H69AR and H446/H446DDP cells. Scale bars, 25 μm. Quantification of DCP2 expression detected by immunofluorescence is shown. The data originated from 3 independent experiments. **P < 0.01; ***P < 0.001.


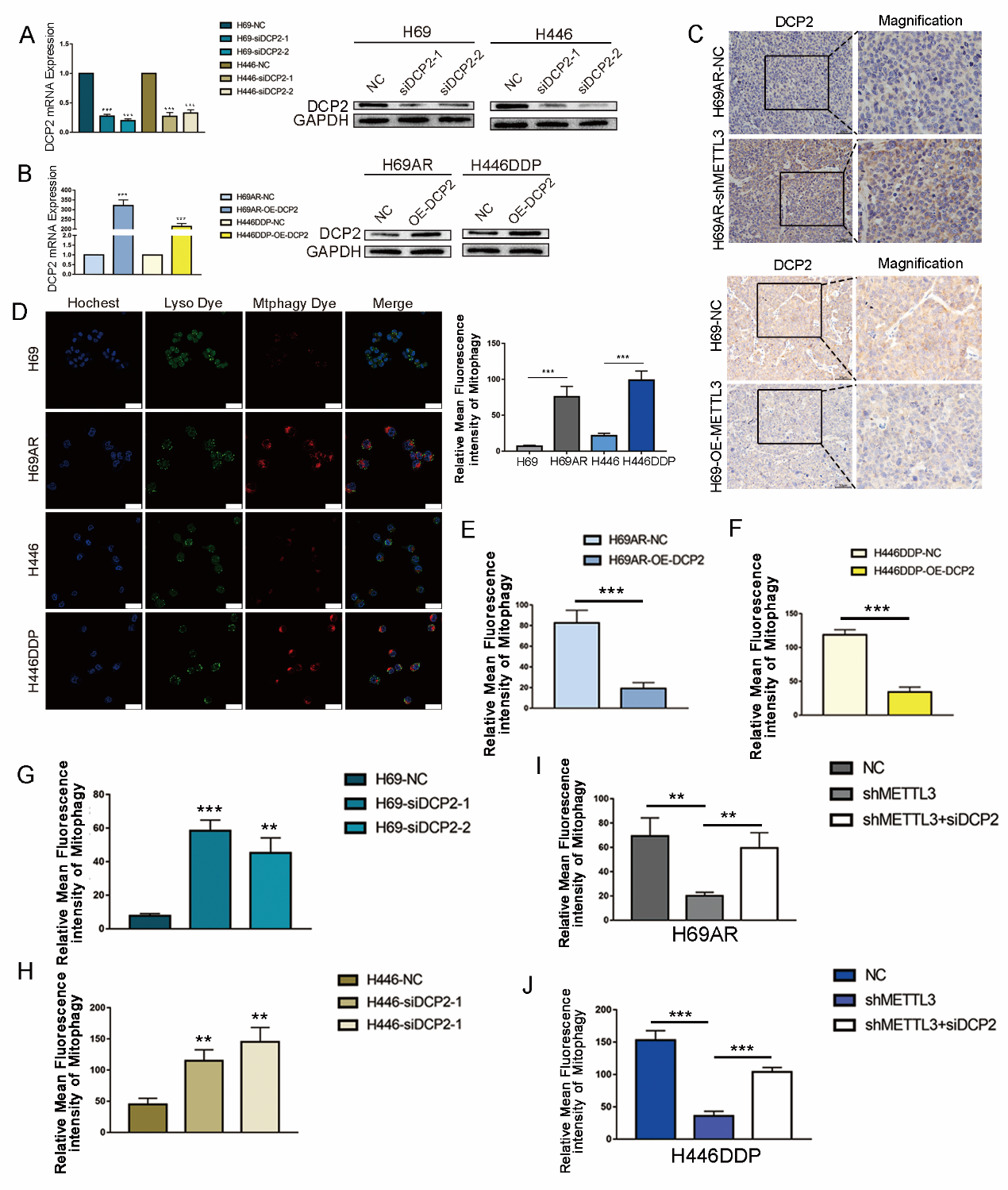


**Figure S3. DCP2 prevents SCLC chemoresistance by regulating mitophagy. (A)** Knockdown of DCP2 by transfection of DCP2 siRNA in H69 and H446 cells. ***P < 0.001. **(B)** Overexpression of DCP2 using plasmid in H69AR and H446DDP cells. ***P < 0.001. **(C)** Representative IHC staining for DCP2 in subcutaneous xenografts with or without METTL3 knockdown. Scale bars, 50 μm. **(D)** Mtphagy images show mitophagy fluorescence, and merged images show colocalization of mitochondrial and lysosomal dyes in two pairs of chemosensitive and chemoresistant SCLC cell lines after chemotherapeutic drug treatment. The right histogram shows the quantified mitophagy signal. Scale bar represents 25 μm. ***P < 0.001. **(E-J)** The histograms show the quantified mitophagy signal. **P < 0.01; ***P < 0.001.


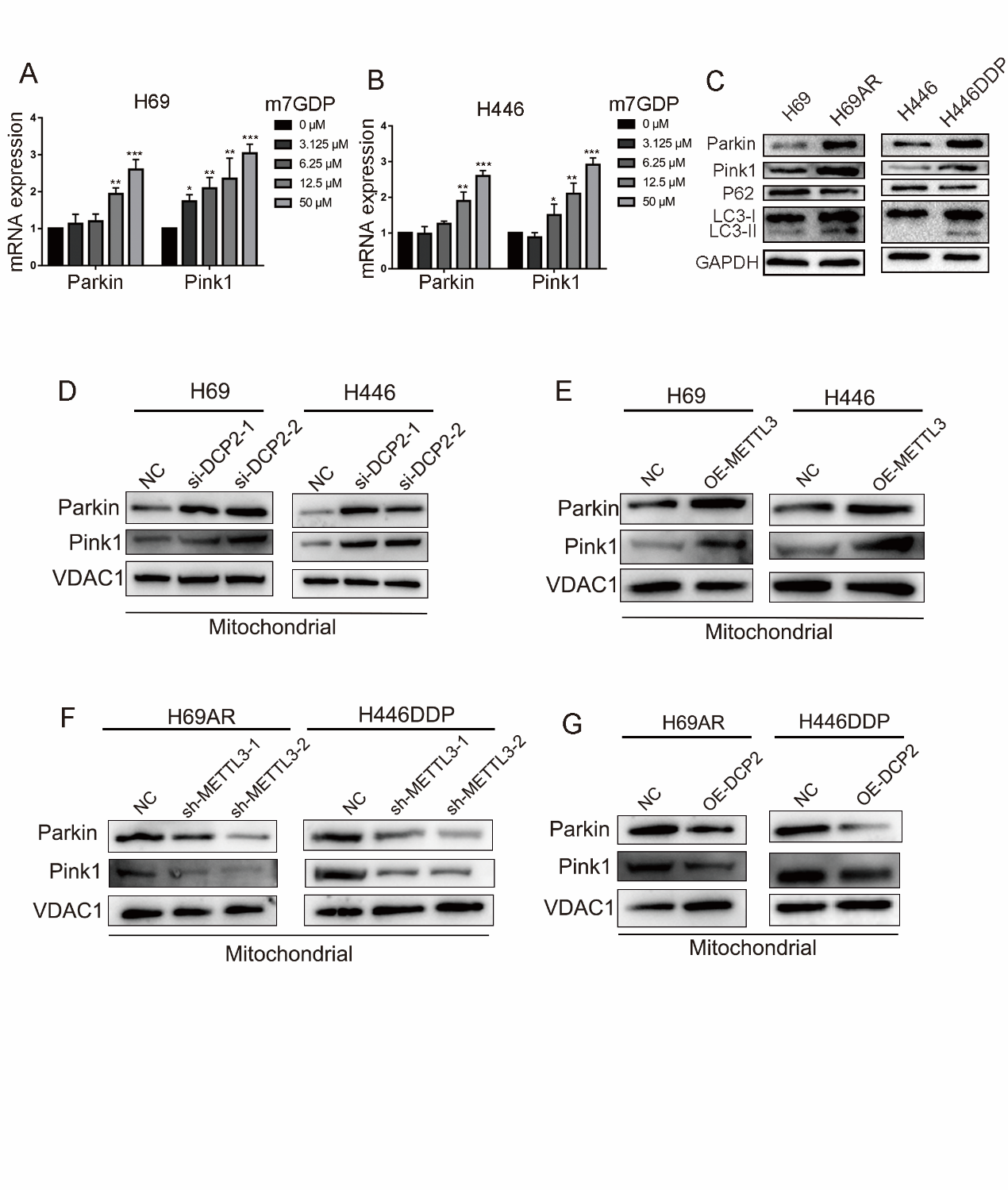


**Figure S4. DCP2 regulates the Pink1-Parkin pathway.** **(A-B)** qRT‐PCR analysis of the mRNA expression of Pink1 and Parkin in chemosensitive cells treated with m7GDP at different concentrations. *P < 0.05, **P < 0.01; ***P < 0.001. **(C)** Western blot analysis of mitophagy-related protein expression in two pairs of chemosensitive and chemoresistant SCLC cells after chemotherapeutic drug treatment. **(D-E)** Western blot analysis of Parkin and Pink1 protein expression in mitochondria after knockdown of DCP2 (D) and overexpression of METTL3 (E) in the drug-sensitive cell lines H69 and H446. **(F-G)** Western blot analysis of Parkin and Pink1 protein expression in mitochondria after knockdown of METTL3 (F) and overexpression of DCP2 (G) in the drug-resistant cell lines H69AR and H446DDP.


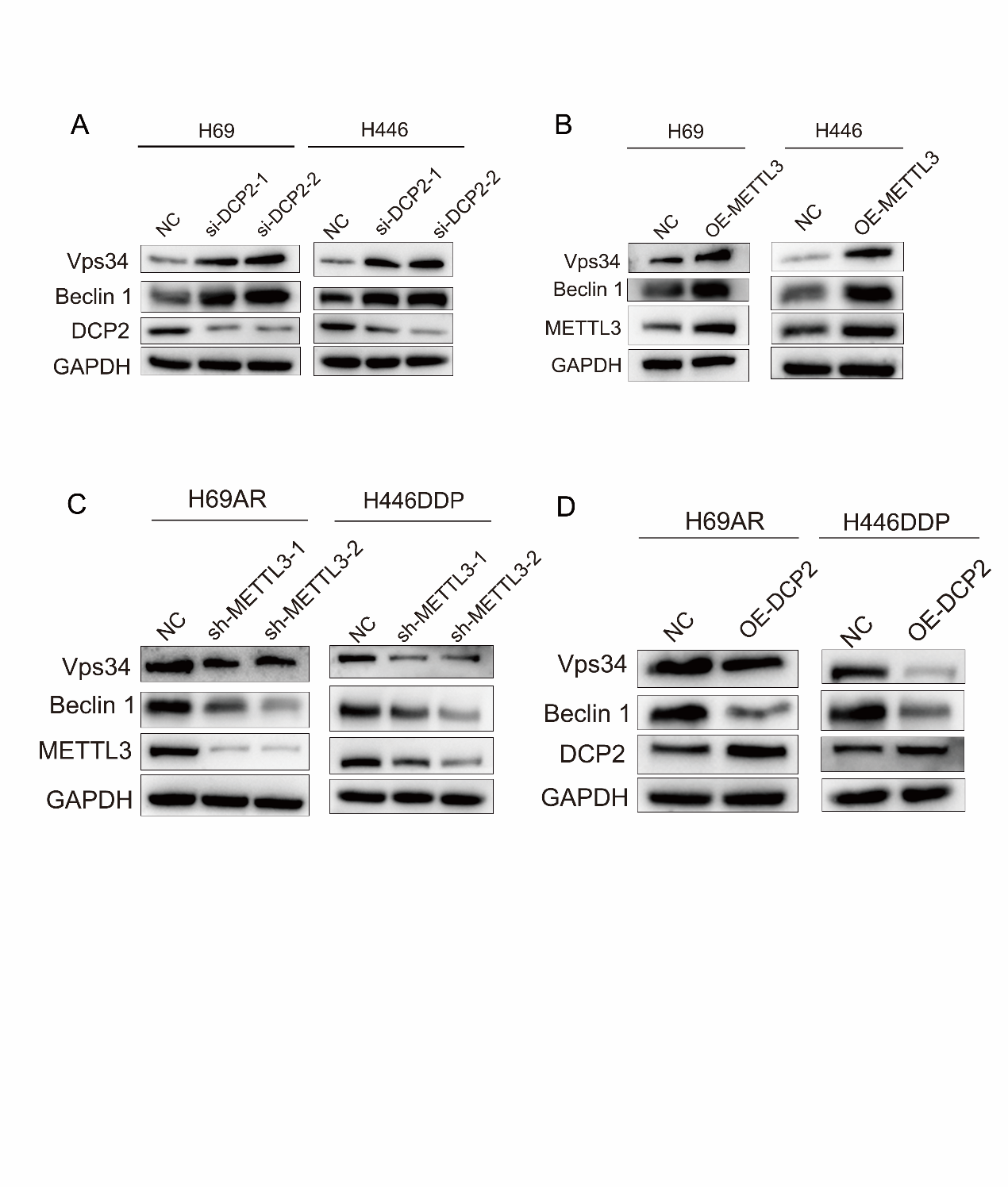


**Figure S5. METTL3 and DCP2 regulate the expression of the autophagy-related proteins Beclin 1 and Vps34. (A-B)** Western blot analysis of Beclin 1 and Vps34 protein expression after knockdown of DCP2 (A) and overexpression of METTL3 (B) in the drug-sensitive cell lines H69 and H446. **(C-D)** Western blot analysis of Beclin 1 and Vps34 protein expression after knockdown of METTL3 (C) and overexpression of DCP2 (D) in the drug-resistant cell lines H69AR and H446DDP.


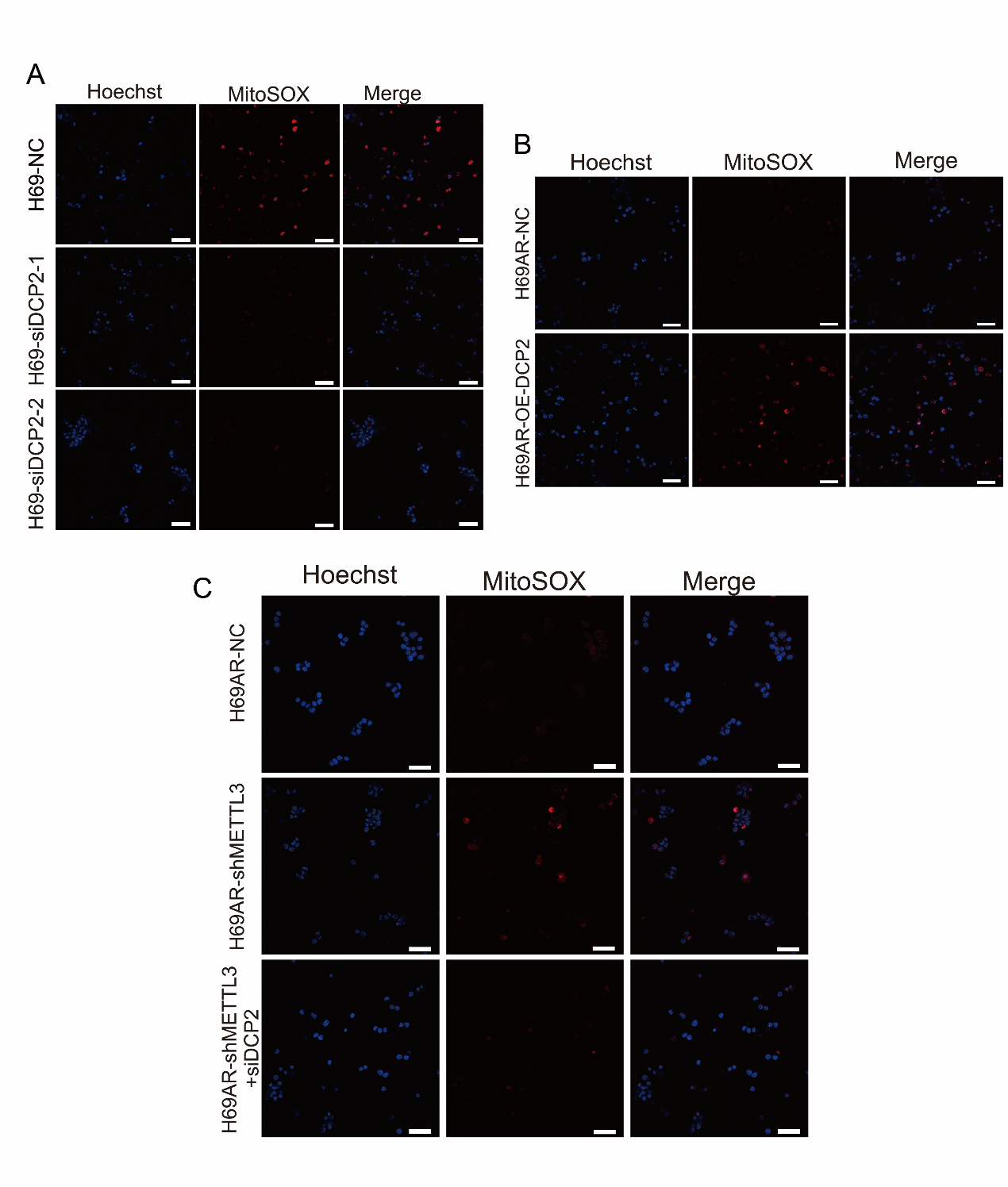


**Figure S6. DCP2 regulates mitochondrial damage levels in SCLC cells. (A)** MitoSOX levels were observed by confocal microscopy in DCP2-knockdown SCLC cells following treatment with chemotherapeutic drugs. Scale bar: 50 μm. **(B)** MitoSOX levels were observed by confocal microscopy in DCP2-overexpressing SCLC cells following treatment with chemotherapeutic drugs. Scale bar: 50 μm. **(C)** MitoSOX levels were observed by confocal microscopy in SCLC cells with simultaneous knockdown of METTL3 and DCP2 following treatment with chemotherapeutic drugs. Scale bar: 50 μm.**
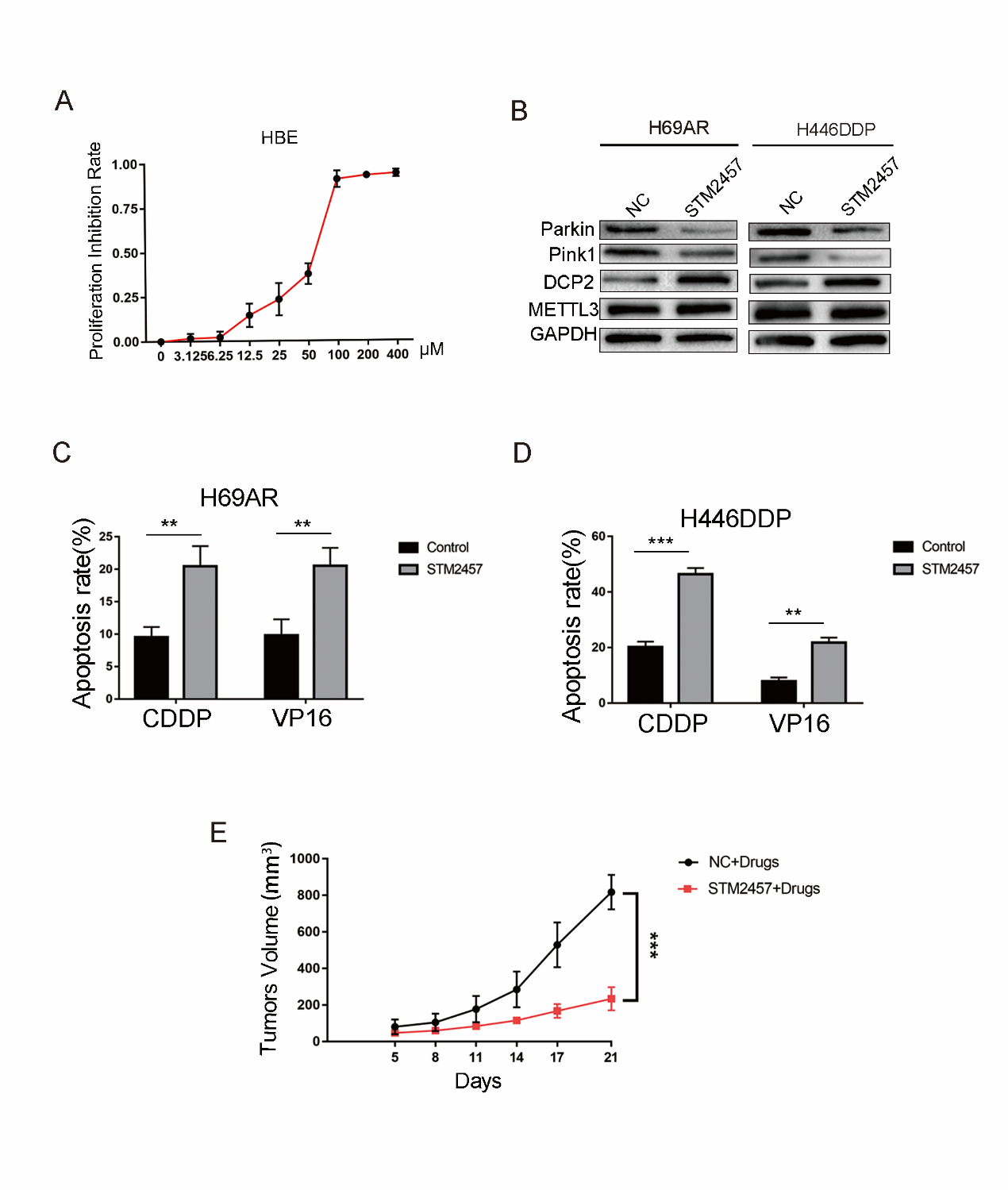
 Figure S7. The METTL3 inhibitor STM2457 reverses chemoresistance in SCLC cells. (A)** CCK-8 assay showing the proliferation of normal lung epithelial HBE cells at different concentrations of STM2457. **(B)** Western blot analysis of mitophagy-related protein expression after STM2457 treatment (6.25 μM) in the drug-resistant cell lines H69AR and H446DDP. **(C-D)** Bar graph showing that STM2457 significantly increased the proportion of apoptotic cells in chemotherapy-resistant cell lines **P < 0.01; ***P < 0.001. **(E)** Tumour growth measurement in the STM2457-treated groups. H69AR cells were incubated for 24 hours with or without STM2457 (6.25 μM) and then injected subcutaneously into nude mice, and chemotherapy drugs or vehicles were administered intraperitoneally. ***P < 0.001.
